# Two light sensors decode moonlight versus sunlight to adjust a plastic circadian/circalunidian clock to moon phase

**DOI:** 10.1073/pnas.2115725119

**Published:** 2022-05-27

**Authors:** Martin Zurl, Birgit Poehn, Dirk Rieger, Shruthi Krishnan, Dunja Rokvic, Vinoth Babu Veedin Rajan, Elliot Gerrard, Matthias Schlichting, Lukas Orel, Aida Ćorić, Robert J. Lucas, Eva Wolf, Charlotte Helfrich-Förster, Florian Raible, Kristin Tessmar-Raible

**Affiliations:** ^a^Max Perutz Labs, University of Vienna, 1030 Vienna, Austria;; ^b^Research Platform “Rhythms of Life", University of Vienna, 1030 Vienna, Austria;; ^c^Department for Neurobiology and Genetics, Theodor-Boveri Institute, Biocentre, University of Würzburg, 97074 Würzburg, Germany;; ^d^Institute of Molecular Biology, 55128 Mainz, Germany;; ^e^Institute of Molecular Physiology, Johannes Gutenberg-University of Mainz, 55128 Mainz, Germany;; ^f^Division of Neuroscience & Experimental Psychology, University of Manchester, Manchester M13 9PT, United Kingdom;; ^g^Howard Hughes Medical Institute, Brandeis University, Waltham, MA 02454;; ^h^Alfred Wegener Institute, Helmholtz Centre for Polar and Marine Research, 27570 Bremerhaven, Germany;; ^i^Carl-von-Ossietzky University, 26111 Oldenburg, Germany

**Keywords:** molecular clock, moon light, chronobiology, reproduction, marine biology

## Abstract

Many species synchronize their physiology and behavior to specific hours. It is commonly assumed that sunlight acts as the main entrainment signal for ∼24-h clocks. However, the moon provides similarly regular time information. Consistently, a growing number of studies have reported correlations between diel behavior and lunidian cycles. Yet, mechanistic insight into the possible influences of the moon on ∼24-h timers remains scarce. We have explored the marine bristleworm *Platynereis dumerilii* to investigate the role of moonlight in the timing of daily behavior. We uncover that moonlight, besides its role in monthly timing, also schedules the exact hour of nocturnal swarming onset to the nights’ darkest times. Our work reveals that extended moonlight impacts on a plastic clock that exhibits <24 h (moonlit) or >24 h (no moon) periodicity. Abundance, light sensitivity, and genetic requirement indicate that the *Platynereis* light receptor molecule r-Opsin1 serves as a receptor that senses moonrise, whereas the cryptochrome protein L-Cry is required to discriminate the proper valence of nocturnal light as either moonlight or sunlight. Comparative experiments in *Drosophila* suggest that cryptochrome’s principle requirement for light valence interpretation is conserved. Its exact biochemical properties differ, however, between species with dissimilar timing ecology. Our work advances the molecular understanding of lunar impact on fundamental rhythmic processes, including those of marine mass spawners endangered by anthropogenic change.

## Significance

The moon provides highly reliable time information to organisms. Whereas sunlight is known to set daily animal timing systems, mechanistic insight into the impact of moonlight on such systems remains scarce. We establish that the marine bristleworm *Platynereis dumerilii* times the precise hours of mass spawning by integrating lunar light information into a plastic daily timing system able to run with circadian (∼24 h) or circalunidian (∼24.8 h) periodicity. The correct interpretation of moonlight is mediated by the interplay of two light sensors: a cryptochrome and a melanopsin ortholog provide information on light valence and moonrise time, respectively. Besides its ecological relevance, our work provides a plausible explanation for long-standing observations of light intensity–dependent differences in circadian clock periods.

## A Moonlight-Sensitive Clock Times Swarming Behavior

**Fig. 1.**
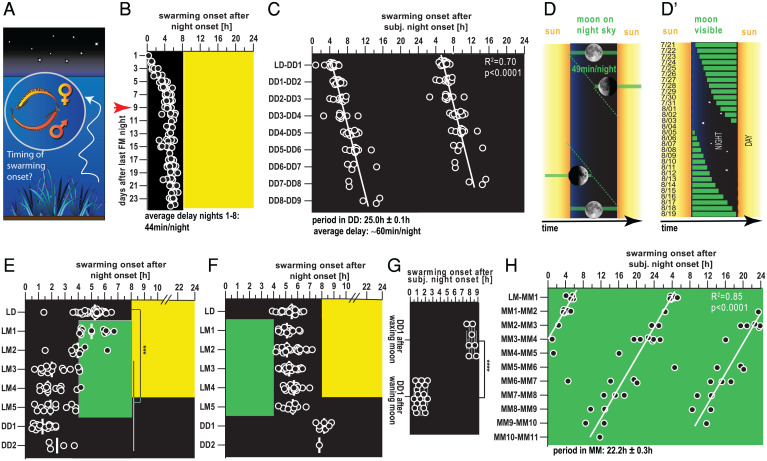
A moonlight-sensitive PCC clock times swarming onset to darkness. (*A*) Schematized swarming behavior of *P. dumerilii*. (*B*) Swarming onset of individual, separated worms across different days of an artificial lunar month, where worms receive eight nights of continuous nocturnal light (FM) every month in addition to a 16:8 h LD cycle (for details, see refs. 1, 2). Red arrow indicates from which day of the circalunar cycle onward worms were used for all subsequent experiments (except Fig. 2 *F* and *G*). (*C* and *H*) Swarming onset of worms released into constant darkness (DD; *C*) or constant moonlight (MM; *H*). Data are double-plotted for better visualization. White lines are linear regression lines. Period lengths were calculated based on the slope of the regression line ± the 95% CI of the slope. (*D* and *D′*) Schemes illustrating moonrise and moonset times in a simplified averaged model (*D*) and a natural habitat (Bay of Naples, July and August 1929) (*D′*) (https://www.timeanddate.com/moon/italy/naples and ref. 3). (*E* and *F*) Swarming onset of worms subjected to naturalistic moonlight during the second (*E*) or first (*F*) half of the night (mimicking waning and waxing moonlight regimes, respectively). Black, no light; yellow, naturalistic sunlight; green, naturalistic moonlight. (*G*) Swarming onset during DD1 after a waning moonlight regime (data from *E*) or a waxing moonlight regime (data from *F*). ****P < 0.0001 for unpaired *t* test.

*Platynereis dumerilii* reproduces by nocturnal mass spawning, with sexually mature males and females synchronously rising from seagrass to the water surface (Fig. 1*A*) during the night (3). Whereas it is well established that this swarming is synchronized to specific nights of the month by a circalunar oscillator (1, 4, 5), we reasoned that it should further increase reproductive success if worms synchronized the onset of swarming behavior also to specific hours during those nights. In fact, such an interconnection of different timing systems is well established for polychaete relatives like the palolo worms (6) and fireworms (*Odontosyllis*) (7). Indeed, modulatory effects of the moon on behavior and physiology have been described for various animals (8, 9).

This prompted us to investigate if *P. dumerilii* also exhibits preferred hours of swarming. We placed maturing, monthly (circalunar) entrained *P. dumerilii* adults (5, 10) in individual wells of our automated behavioral recording device (11). As swarming is accompanied by a burst of swimming activity (“nuptial dance”), analysis by automated video tracking allowed us to systematically deduce the time of swarming onset with respect to the daylight/darkness ( light:dark [LD], 16:8 h) cycle (*SI Appendix*, Fig. S1 *A* and *B* and Movie S1). Analyses of 139 individuals revealed that swarming onset across the culture was indeed synchronized to an ∼1- to 2-h window during the night (Fig. 1*B*). (Note that we selected about equal numbers of swarming worms per night. Therefore, the monthly swarming synchronization is invisible.) The precise time point depended on the time since the last artificial full moon (FM) night (Fig. 1*B*), which is provided to entrain the worms’ monthly oscillator (4, 5). In nights directly following the last FM night, animals started the characteristic swarming behavior directly following night onset. This onset of swarming gradually shifted by approximately 44 min per night within the first eight nights (Fig. 1*B*, days preceding the red arrow). For the remaining lunar month, the time of swarming onset remained unaltered at ∼5 h after night onset (Fig. 1*B* and *SI Appendix*, Fig. S1*B*). To assess whether this synchronization was driven by an endogenous oscillator, we next monitored swarming onset in worms that were kept in constant darkness for several days. Under these dark–dark (DD) conditions, swarming was still synchronously initiated, with an average delay of ∼1 ± 0.1 h/d (Fig. 1*C*). This established that the specific hour of nocturnal swarming onset is controlled by an endogenous clock.

The time delay of about 44 min within the first eight nights after FM is reminiscent of the average delay of the rise of the waning moon (∼49 min per night; Fig. 1*D*). This apparent delay of moonrise time relative to sunset is caused by the period difference of the daily solar cycle (24 h) and the lunidian cycle (24.8 h; the average time span between two successive moonrises) (Fig. 1*D*). The latter matches the period length of the endogenous clock (∼25 h) controlling swarming onset under DD conditions (compare Fig. 1 *C* and *D*). The combination of these facts let us speculate that the worm’s ∼24-h timing system could help to synchronize swarming onset to the darkest hours of the night but would require the moon for entrainment. Furthermore, the exact change of moonrise relative to sunset is not always exactly ∼49 min per night but varies under natural conditions (Fig. 1*D′*), making an additional adjustment by moonlight likely advantageous. We thus next studied if the endogenous clock was sensitive to moonlight for its exact entrainment. To mimic moonlight and sunlight under laboratory conditions, we complemented available surface measurements (12) by analyzing systematic light measurements at a natural habitat of *Platynereis* (*SI Appendix*, Fig. S2*A*), which guided the design of naturalistic sunlight and naturalistic moonlight illumination devices (*SI Appendix*, Fig. S2*B*, and see also refs. 1, 11).

We next exposed animals (≥9 d after the end of the monthly nocturnal light stimulus; red arrow in Fig. 1*B*) to naturalistic moonlight (*SI Appendix*, Fig. S2*B*) provided during the second half of the night for five consecutive nights (Fig. 1*E*, LM1 to LM5). In response to this light regime mimicking waning moon, worms shifted their swarming onset gradually into the dark portion of these moonlit nights (Fig. 1*E*). The advanced swarming onset caused by the waning moonlight regime persisted when worms were subsequently released into constant darkness (Fig. 1*E*, DD1 and DD2), arguing that this shift was caused by an impact of moonlight on the endogenous clock, rather than being an acute masking effect (i.e., direct response to light). When the same naturalistic moonlight was provided during the first half of the night (mimicking times of waxing moon), no shift of swarming times was observed (Fig. 1*F*). Comparisons of a subsequent constant darkness period (Fig. 1*F*, DD1 and DD2) showed a significant difference of clock-controlled swarming onset compared to the waning moonlight regime (Fig. 1*G*). Finally, under a constant naturalistic moonlight (MM) regime, swarming onset remained synchronized but occurred with a period length of ∼22.2 ± 0.3 h (Fig. 1*H*), a clear period shortening compared to DD conditions (Fig. 1*C*).

Taken together, these results suggest the existence of a plastic oscillator system that regulates nocturnal swarming onset. In the absence of a moonlight stimulus it free-runs during the first nine nights after FM. Naturalistic moonlight mimicking the full or waning moon modulates the clock’s period and/or resets the clock’s phase. This results in a swarming preference during the dark portion of the night, consistent with natural observations. We refer to this clock as plastic circadian/circalunidian (PCC) clock.

## L-Cry Is Required to Correctly Interpret Sunlight and Moonlight to Set the Swarming Hour

In order to understand how (naturalistic) sunlight and moonlight are sensed and distinguished by this system, we next sought to identify photoreceptor(s) relevant for the light impact on the PCC clock. One candidate receptor of particular interest was *Platynereis* L-Cryptochrome (L-Cry). Based on expression changes, a distant homolog (Cry2) in the coral *Acropora* has been speculated to mediate moonlight sensation (13). In a separate study, we uncovered that *Pdu-*L-Cry has the biochemical and cellular properties to discriminate between sunlight and moonlight and is required to correctly detect the FM phase for monthly oscillator entrainment (1).

**Fig. 2.**
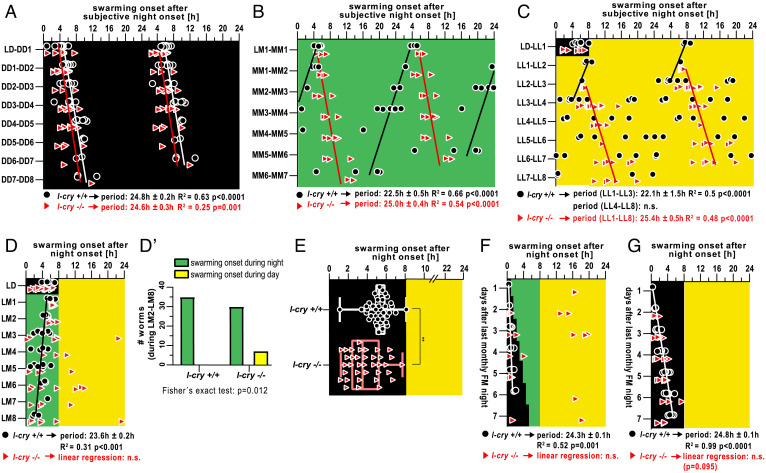
Platynereis L-Cry enables the PCC clock, a clock with ∼24 h period, to distinguish sunlight versus moonlight. (*A*–*C*) Swarming onset of *l-cry^−/−^* mutants (red triangles) and wild types (black circles) entrained to 16:8 h LD cycles and subsequently released into (*A*) constant darkness (DD), (*B*) constant naturalistic moonlight (MM), or (*C*) constant naturalistic sunlight (LL). Data are double-plotted. Black and red lines indicate linear regression lines of wild-type and *l-cry^−/−^* mutants, respectively. The period length was calculated based on the slope of the regression line ± the 95% CI of the slope. Data of *l-cry^+/+^* worms in *B* and *G* are also shown in Fig. 1 *H* and *B*, respectively. (*D*) Swarming onset of *l-cry^−/−^* mutants and wild types entrained to 16:8 h LD cycles and subsequently subjected to alternations of naturalistic sunlight during the day and moonlight during the night (LM). (*D′*) Number of *l-cry^−/−^* mutants that initiate swarming onset during the day under the LM light regime (data LM2 to LM8 from *D*) is significantly increased. (*E*) Swarming onset of *l-cry^−/−^* mutants and wild types maintained under 16:8 h LD cycles (**P = 0.004, F-test to test if the variances in the two groups are significantly different). (*F* and *G*) Swarming onset of *l-cry^−/−^* mutants and wild types assessed directly after the monthly nocturnal FM light stimulus with either an additional waning moonlight regime (*F*) or kept under LD cycles (*G*).

To assess if *Platynereis* L-Cry is also relevant for the light input into an oscillator with the period length of ∼24 h, the PCC clock, we analyzed a *Platynereis l-cry* loss-of-function strain generated by Transcription activator-like effector nucleases (TALEN) technology (for details on mutants, see ref. 1). When exposed to constant darkness, *l-cry^−/−^* individuals still exhibited rhythmic initiation of swarming onset, with a period length (24.6 ± 0.3 h) indistinguishable from wild types (Fig. 2*A*). This indicates that L-Cry is not required for the endogenous oscillation of the PCC clock.

To probe for roles of L-Cry in mediating light input into the PCC clock, we next investigated swarming rhythmicity in *l-cry^–/–^* mutants exposed to constant naturalistic moonlight (MM) or naturalistic sunlight (LL). Under both conditions, *l-cry^–/–^* mutants exhibited a synchronized swarming onset, with period lengths (MM, 25 ± 0.4 h [Fig. 2*B*]; LL, 25.4 ± 0.5 h [Fig. 2*C*]) highly reminiscent of the period of wild type in DD conditions (Fig. 2*A*). In contrast, wild-type siblings shortened their period (MM) or became arrhythmic (LL) (Fig. 2 *B* and *C*). These clear differences between wild type and mutants let us conclude that L-Cry is relevant for the conveying of naturalistic sunlight and moonlight information to the PCC clock.

The absent adjustment of the PCC clock in *l-cry^−/−^* individuals to respond to light could be explained by a general reduction in light sensitivity. Alternatively, these findings are compatible with a role of L-Cry in distinguishing moonlight and sunlight, as L-Cry enables the PCC clock to respond differently to the two light conditions and as it also possesses this property in the context of the worm’s monthly oscillator (for monthly oscillator, see ref. 1). To discriminate between the two possibilities, we exposed *l-cry* mutants to a combined naturalistic day/night light regime of 16:8 h, where they were exposed to naturalistic sunlight during the day and naturalistic moonlight during the entire night, mimicking the FM situation (light:moonlight [LM]) (Fig. 2*D*). If *l-cry^−/−^* animals were simply blind to light, they should continue to exhibit the swarming timing seen in Fig. 2 *B* and *C*. However, if *l-cry^−/−^* rather provided interpretation on the nature of the light stimulus to other photoreceptors, the prediction was that this would cause an increased behavioral variability between individual worms as critical light valence information is missing in a mixed naturalistic sunlight/moonlight regime. Indeed, unlike wild-type animals, which restricted swarming onset strictly to nocturnal hours (Fig. 2 *D* and *D′*), and different from the timing observed under constant moonlight (Fig. 2*B*) or constant sunlight (Fig. 2*C*), *l-cry^–/–^* mutants exhibited aberrant, much more variable swarming onset timing under the complex naturalistic sunlight and moonlight regime (Fig. 2*D*), with a significant proportion (∼19%) of *l-cry^−/−^* worms initiating swarming during the day (Fig. 2*D′*). Furthermore, and also consistent with L-Cry’s valence function, all *l-cry^–/–^* mutants restricted swarming onset to the night when no moonlight was present, albeit slightly less synchronized and earlier than wild type (Fig. 2 *D* [see LD before LM] and Fig. 2*E*). This further supports that the shifted timing into the day was caused by the naturalistic moonlight stimulus, which got misinterpreted by the *l-cry^−/−^* animals. Our findings indicate that the *l-cry* mutation does not simply render worms less sensitive to moonlight, but that L-Cry is required to provide the correct light valence information (valence is discrimination of sunlight versus moonlight based on intensity and/or spectrum) to the PCC clock. Furthermore, our data are consistent with the idea that constant moonlight during the night (i.e., FM) is used to reset the hour of swarming to the early night hours observed directly after FM, likely via modulating the period length of the PCC clock.

In an extended nontiled waning moonlight regime (as used in Fig. 1*E*), *l-cry^−/−^* mutants and wild-type individuals swarmed similarly (*SI Appendix*, Fig. S3*A*), except that *l-cry^−/−^* mutants initiated swarming again already slightly earlier during the LD cycle (*SI Appendix*, Fig. S3*A* and compare Fig. 2 *D* and *E*). By contrast, an abnormal (confused) swarming onset of *l-cry^−/−^* animals similar to the FM situation (Fig. 2*D*) was observed in a light regime in which a tiled, waning moonlight stimulus (*SI Appendix*, Fig. S2*C*) was provided directly following the standard monthly culture FM stimulus (Fig. 2*F*). The tiled moonlight regime more closely mimics the natural timing of moonlight during nights in which swarming is observed (Fig. 2*F* and compare Fig. 1 *D* and *D′*). Under a corresponding LD regime lacking the moonlight stimulus, the abnormal swarming onset of the mutants was not observed (Fig. 2*G*).

We reason that the phenotypic difference between the nontiled and tiled waning moonlight regimes is likely due to the ability of worms to adjust their circadian output also to the solar photoperiod (a common phenomenon in various animals). Indeed, an equivalent long photoperiod has comparable effects (*SI Appendix*, Fig. S3*B*). Thus, even an unclear interpretation of light by *l-cry^–/–^* mutant worms results in a similar behavioral output. Likely *l-cry^–/–^* mutants misinterpreted the moonlight stimulus in a nontiled waning regime as a long photoperiod (*SI Appendix*, Fig. S3 *A* and *B*). By contrast, the more naturalistic tiled waning moonlight regime creates similarly abnormal swarming times in *l-cry^–/–^* mutants, as does the naturalistic FM regime. This emphasizes the importance of naturalistic light regimes for functional light receptor studies. It further confirms the light discriminatory role of L-Cry, which is especially relevant under more naturalistic conditions.

## Evidence for Distinct Signaling of L-Cry under Sunlight versus Moonlight

In the common view based on the work in *Drosophila melanogaster*, the fly 1:1 ortholog of L-Cry—dCRY (14)—undergoes light-dependent binding to Timeless, which leads to the degradation of both Timeless and dCRY, thus resetting the flies’ circadian clock upon light input (reviewed in ref. 15). This binary signaling model is difficult to reconcile with our finding that in the adjustment of the *Platynereis* PCC clock, L-Cry is relevant for the distinction between moonlight versus sunlight, characterized by different irradiance levels and spectra.

**Fig. 3.**
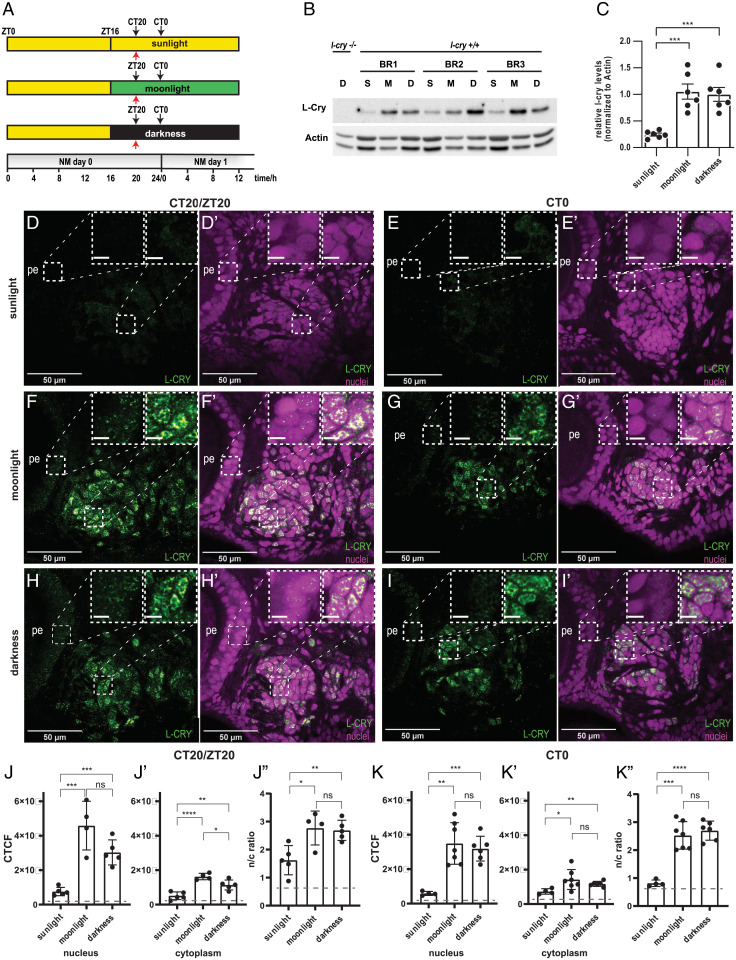
*Pdu-*L-Cry abundance and localization under darkness, naturalistic sunlight, and moonlight. (*A*) Sampling scheme of *Platynereis* heads for Western blot and immunohistochemistry. Red arrows indicate Western blots. Black arrows indicate immunohistochemistry. (*B* and *C*) Head extracts sampled under naturalistic sunlight (S), moonlight (M), and darkness (D) were analyzed by Western blot (representative blot of three biological replicates [BR1-3] shown) and normalized against beta-actin, *n* = 6 BRs. Bar graph shows mean ± SEM. (*D*–*I′*) Wild-type worm heads sampled under indicated naturalistic sunlight, moonlight, and darkness conditions, stained for *Pdu*-L-Cry (green) and including nuclei stained with HOECHST (violet). (Scale bar in *Insets*, 5 µm.) For a subsequent time point at CT12, see *SI Appendix*, Fig. S4. (*J*–*K″*) Quantification of immunofluorescent images, with a threshold (gray) indicating the mean value of immunofluorescence in *l-cry^−/−^* mutants. *P < 0.05; **P < 0.01, ***P < 0.001; ****P < 0.0001 for unpaired *t* test; ns: not significant.

Furthermore, we had observed that under conditions relevant for monthly oscillator entrainment, L-Cry’s protein levels and subcellular localization markedly differ between naturalistic sunlight versus moonlight conditions (1). As the conditions relevant for the PCC clock entrainment are different from those relevant for the monthly oscillator entrainment, we therefore tested if L-Cry protein in the worm exhibited any differences when animals were exposed to naturalistic sunlight or moonlight under conditions relevant for the behavioral paradigms shown in Figs. 1 and 2 (and see Fig. 6) and *SI Appendix*, Figs. S3 and S7. For this, we made use of a *Pdu-*L-Cry–specific antibody (1). We first assessed L-Cry abundance in head extracts of animals sampled at the midpoint of the subjective night (at new moon), after 4 h of darkness or exposure to either naturalistic sunlight or moonlight (Fig. 3 *A*, CT20, red arrows). As expected by the canonical *Drosophila* model, and consistent with our previous analyses in S2 cells (5), naturalistic sunlight led to a significant reduction of L-Cry compared to heads sampled from animals maintained in darkness (Fig. 3 *B* and *C*). In contrast, the levels of L-Cry protein in the heads of naturalistic moonlight-exposed animals were indistinguishable from dark levels (Fig. 3 *B* and *C*).

Immunohistochemical analyses at two distinct time points of the respective light regime (ZT/CT20; CT0, black arrows in Fig. 3*A*) and the following midday point (CT12, black arrows in *SI Appendix*, Fig. S4*A*) revealed that L-Cry was predominantly localized in the nuclei of the eye photoreceptors and of cells in the posterior oval-shaped brain domain under naturalistic moonlight (Fig. 3 *F*–*G′* and *SI Appendix*, Fig. S4 *C* and *C′* and *Insets*; for comparison to light/dark conditions, see Fig. 3 *H*–*I′* and *SI Appendix*, Fig. S4 *D* and *D′*; quantifications in Fig. 3 *J*–*K″*). By contrast, continuous exposure to naturalistic sunlight resulted in very low but still detectable levels with similar distributions in both nucleus and cytoplasm, resulting in an altered nuclear/cytoplasmic ratio (Fig. 3 *D*–*E′*, Insets; *SI Appendix*, Fig. S4 *B* and *B′*, and quantifications in Fig. 3 *J*–*K″*). The very low L-Cry levels we detect here under continuous naturalistic sunlight, together with our previous analyses (1, 5), support the notion that a degradation pathway is triggered by sunlight but not moonlight. In combination with the behavioral phenotypes, this further strengthens the concept that L-Cry is required for the correct interpretation of sunlight versus moonlight for the PCC clock.

## Pharmaceutical Disruption of Canonical Core Circadian Clock Oscillations Affects the PCC Clock

We next wondered whether the PCC clock required the activity of the conventional core circadian clock. We previously showed that an inhibitor of casein kinase 1δ/ε, PF670462, disrupts the worms’ core circadian clock gene oscillations (5). The effect of this drug on the core circadian clock has also been shown in several other aquatic animals, as diverse as cnidarian, crustacean, and teleost fish species (16–18).

After validating that an incubation in 160 nM of PF670462 abolished molecular oscillations of core circadian clock transcripts (*SI Appendix*, Fig. S5*A*), we assessed the effects of the drug on the timing of swarming onset. In contrast to mock-treated controls, the swarming onset in constant darkness was disrupted upon drug treatment (*SI Appendix*, Fig. S5*B*). This finding is consistent with the notion that at least a subset of canonical circadian clock genes is required for the PCC clock, although we can at present not rule out that this effect could be caused by other targets of casein kinase 1δ/ε.

## dCRY Prevents the Fly’s Circadian Clock from Misinterpreting Moonlight

As a regular nocturnal stimulus, moonlight reaches aquatic and terrestrial habitats. The ability to properly discriminate between moonlight and sunlight is therefore likely important for any species that uses light-sensitive clocks. In many species, the conventional circadian clock should likely run with a constant period, irrespective of lunar phase. Thus, moonlight would need to be blocked from interfering with circadian rhythmicity in those organisms. Indeed, whereas fruit fly circadian behavior can be experimentally entrained to LD cycles with light below FM light intensity (19, 20), and constant light at moonlight intensity can extend the period length of wild-type flies (21, 22), moonlight does not cause major effects on the circadian clock when combined with an LD cycle in this species (23–26).

**Fig. 4.**
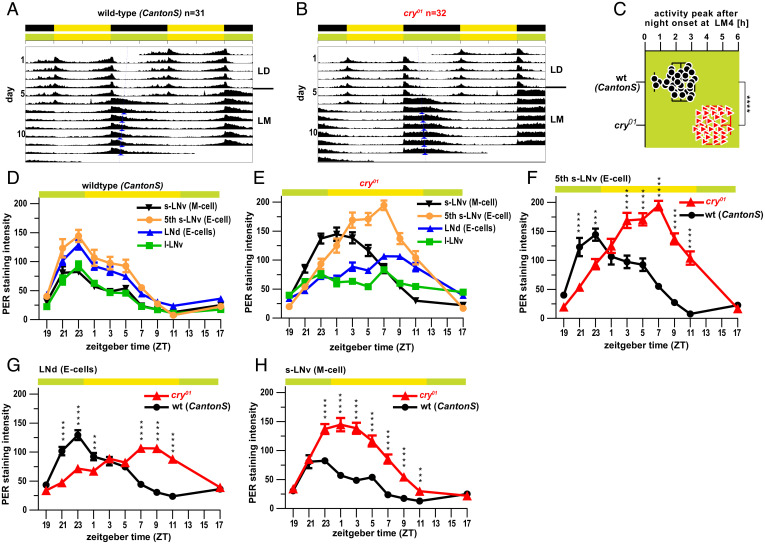
*Drosophila* cry protects circadian oscillator synchrony against moonlight. (*A* and *B*) Double-plotted actograms depicting average activity of wild-type (*A*) and *cry^01^* (*B*) flies subjected to 12:12 h LD cycles followed by LM cycles. Blue arrowheads indicate acrophases of the respective activity rhythms. (*C*) Timing of the E peak during LM4, calculated from the data shown in *A* and *B*. The value 0 represents the time of lights off. (*D* and *E*) Quantified anti-PER immunolabeling intensity in different groups of lateral circadian clock neurons under LM conditions (LM4) in wild-type (*D*) and *cry^01^* (*E*) individuals. (*F* and *G*) Detailed comparison of PER oscillations for neurons controlling evening activity reveals a pronounced phase delay of about ∼8 h in cry^01^ mutants, whereas (*H*) neurons controlling morning activity show a more modest phase delay (2 to 4 h). Data in *F*–*H* are replotted from *D* and *E*. ***P < 0.001; ****P < 0.0001 for ANOVA followed by Sidak’s multiple comparison test.

Given our results about the importance of *Platynereis* L-Cry in discriminating between naturalistic sunlight versus moonlight and *Drosophila* dCRY being its direct 1:1 ortholog, we hypothesized that this principal functionality of the d/L-Cry family might also be present in *D. melanogaster*. Specifically, we wondered if nocturnal light mimicking moonlight would cause an increased shift of the circadian clock in *cry* mutant flies compared to controls. We monitored locomotor behavior of both cantonized *cry^01^* (27) and Canton-S wild-type flies under LM conditions, adapting an existing locomotor paradigm (28) and using an artificial moonlight source matching FM light intensities measured on land (*SI Appendix*, Fig. S2 *D* and *E*). In wild-type flies, moonlight delayed the evening peak to 2.2 ± 0.13 h (mean ± SEM) after night onset (Fig. 4 *A* and *C*), in line with previous observations (24), whereas *cry^01^* mutants exhibited a significantly stronger delay, with the evening activity peak shifting to 4.4 ± 0.11 h (mean ± SEM) after night onset (Fig. 4 *B* and *C*). In comparison, no difference in evening peak activity was observed when wild-type versus *cry^01^* mutants were shifted from LD to DD (*SI Appendix*, Fig. S6*A*). Consistent with our hypothesis, the difference was instead clearly visible between the same wild-type versus *cry^01^* mutant flies under MM (*SI Appendix*, Fig. S6 *B* and *C*).

The increased delay of the evening activity peak in *cry^01^* mutants versus wild type under moonlight could be caused either by acute effects of artificial moonlight on behavior or by a shift in the fly’s circadian clock. In order to discriminate between these possibilities, we subjected flies to artificial LM conditions and used an established immunolabeling strategy to systematically assess, over 10 distinct time points, changes in the abundance of the core circadian clock protein Period (PER) in the lateral neurons harboring the fly’s circadian pacemaker. Anatomical location and the presence or absence of immunoreactivity against the neuropeptide PDF allowed us to quantify Period abundance in l-LN_v_s and s-LN_v_s (below also referred to as morning [M] cells), as well as fifth s-LN_v_s and LN_d_s (clusters harboring the evening [E] cells) (Fig. 4 *D*–*H*).

Quantification across 132 Canton-S wild-type individuals exposed to LM conditions revealed that oscillations of Period protein levels in the different subclusters were in synchrony with each other (Fig. 4*D*). In contrast, the corresponding *cry^01^* mutants exhibited pronounced desynchronization of Period protein oscillations between cell groups, with E cells differing from M cells by ∼6 h (Fig. 4*E*). Similar analyses of *cry^01^*-mutant flies raised in various LD cycles have not revealed such desynchronization (29), indicating that the effects we observed were specifically caused by exposure to artificial moonlight. When comparing Period protein abundances for the different cell classes between *cry^01^* mutants and wild types, Period levels in E cells exhibited a stronger peak delay (∼8 h; Fig. 4 *F* and *G*) than M cells (∼2 h; Fig. 4*H*). This correlates with the fact that the peak of evening activity is significantly delayed in our behavioral analyses of *cry^01^* mutants compared to wild types under LM (Fig. 4 *A* and *B*). It should be noted that the time of fly evening activity is determined by the integrated action of all E cells including the dorsal E neurons (DN_1_-E) (30) that we have not assessed in our study. The DN_1_-E are closely coupled to s-LN_v_s in dark conditions and may have an earlier phase than the lateral E neurons (fifth s-LN_v_ and LN_d_) even in moonlight conditions. This may explain why the behavioral phase shift in evening activity does not completely match the large phase delay observed in the fifth s-LN_v_ and LN_d_.

Taken together, these results indicate that the increased delay of the evening activity peak in cry*^01^* mutants under an LM light regime is the result of a desynchronization of the circadian clock rather than an acute light effect. This suggests that *Drosophila* dCRY is naturally required to reduce the effects of moonlight on circadian clock oscillations, in particular in the cell clusters harboring the evening oscillator.

## L-Cry, but Not dCRY, Is Highly Sensitive to Moonlight

**Fig. 5.**
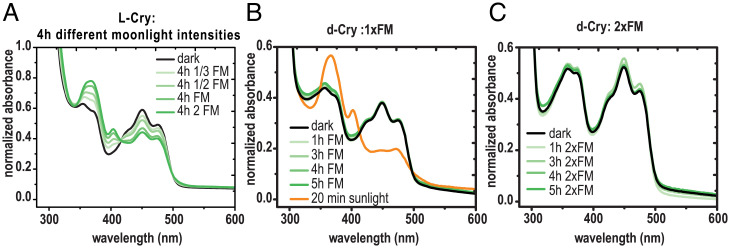
Comparison of L-Cry and dCRY light detection. (*A*) Illumination of purified L-Cry protein with different moonlight intensities (green) for 4 h results in photoreduction (FAD°^−^ formation). FM, naturalistic FM intensity (9.7 × 10^10^ photons/cm^2^/s); 1/3 FM, one third of FM intensity; 1/2 FM, one half of FM intensity; 2 FM, double of FM intensity. (*B* and *C*) dCRY stimulation by moonlight (green) with naturalistic FM intensity (*B*) or double FM intensity (*C*) does not result in photoreduction, whereas naturalistic sunlight (orange) does. For detailed analyses on *Pdu*-L-Cry responses to naturalistic sun and moonlight, see ref. 1.

Given the genetic requirement of both L-Cry and dCRY to correctly interpret moonlight under a combined moonlight/sunlight regime, we next wondered if the biochemical light sensitivity of both orthologs was also comparable. For this, we purified both proteins in the presence of their cofactor flavine adenine dinucleotide (FAD) and tested for changes in absorbance after illumination. When light is sensed by dCRY (31) or L-Cry (1), it changes the oxidized FAD to the reduced anionic radical FAD°^−^ form, visible in the proteins’ absorbance spectrum (31). Extending our work on L-Cry’s biochemical features, we find that *Platynereis* L-Cry does not only respond to naturalistic FM light (1) but does this even at intensities corresponding to 30% of FM intensity at 4 to 5 m seawater depths (Fig. 5*A*). In contrast, dCRY completely failed to respond to naturalistic moonlight levels equivalent to—and exceeding—those eliciting responses in *Platynereis* L-Cry (compare Fig. 5*A* with Fig. 5 *B* and *C*). However, dCRY was activated by naturalistic sunlight, reaching complete FAD reduction within 20 min (Fig. 5*B*) as observed for L-Cry (1), underscoring the integrity of the purified dCRY protein and the functionality of the assay.

Even though dCRY’s sensitivity to dim light might be higher in its cellular context (32), this result clearly points at differences in the molecular mechanisms between dCRY and L-Cry functions. We provide further thoughts on this in *Discussion*. On the ecophysiological level, this might be connected to the different meanings that moonlight has as an environmental cue for the daily behavior of flies versus swarming worms: whereas fly circadian biology is likely optimized to buffer against the effect of moonlight, *Platynereis* worms, as shown in Fig. 1, use moonlight to precisely adjust their nocturnal swarming time to a favorable dark time window.

## R-opsin1 Detects Moonrise to Optimize the Time of Swarming Onset

The retention of moonlight sensitivity in *Platynereis l-cry^–/–^* mutants (as evidenced by the different mutant responses under the combined moonlight and sunlight regimes versus no-moonlight regimes; Fig. 2 *D*–*G*) indicated the existence of one or more additional light receptors required for moonlight sensation. We reasoned that the spectral sensitivity of these photoreceptors likely includes the blue-green range, given the relatively high levels of blue-green light in our moonlight measurements (*SI Appendix*, Fig. S2*A*).

**Fig. 6.**
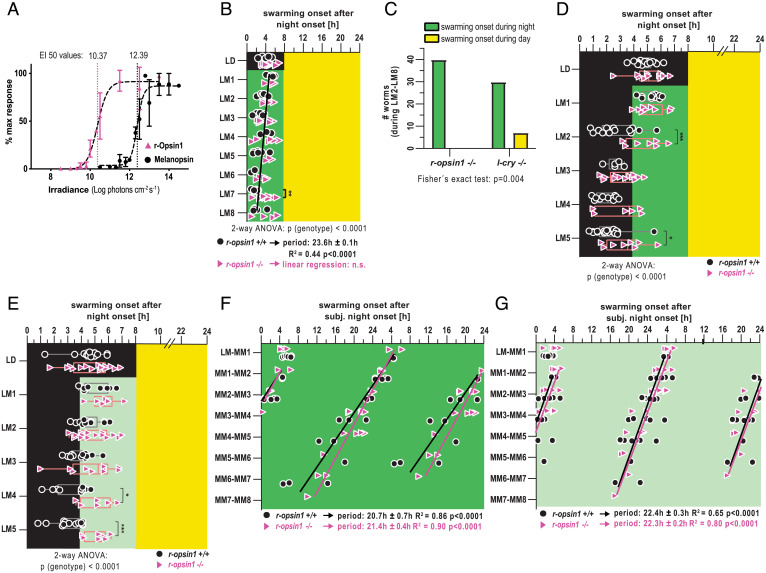
*Pdu*-r-Opsin1 functions as highly light-sensitive photoreceptor to adjust swarming onset to waning moon light. (*A*) Responses of *Pdu-*r-Opsin1 (violet) and human Melanopsin (black) to different blue light intensities (480 nm ± 10 nm), as quantified by a cell-based bioluminescent assay, reveal an ∼100-fold higher sensitivity of Pdu-r-Opsin1. (*B*) Swarming onset of *r-opsin1^−/−^* and *r-opsin1^+/+^* worms entrained to 16:8 h LD cycles and then subjected to constant moonlight of FM light intensity during the night (LM). (*C*) None of the tested *r-opsin1^−/−^* mutants initiated swarming onset during the day under this LM light regime (data LM2 to LM8 from *B*), whereas this number is significantly increased in *l-cry^−/−^* mutants (data LM2 to LM8 from Fig. 2*D*). (*D*–*G*) Swarming onset of *r-opsin1^−/−^* and *r-opsin1^+/+^* worms entrained to 16:8 h LD cycles and then subjected to either moonlight during the second half of the night, mimicking a waning moonlight regime (*D* and *E*), or to constant moonlight (*F* and *G*), either with FM light intensity (dark green) (*B*, *D*, and *F*) or waning moon light intensity (light green indicates 20% of FM light intensity) (*E* and *G*). *P < 0.05; **P < 0.01; ***P < 0.001 for two-way ANOVA followed by Sidak’s multiple comparison test. Black and violet lines in *B*, *F*, and *G* indicate linear regression lines of wild-type and *r-opsin1^−/−^* mutants, respectively. The period length was calculated based on the slope of the regression line ± the 95% CI of the slope.

The gene encoding r-Opsin1 is expressed in the adult *Platynereis* eyes during both early development (33, 34) and later stages (35). In a heterologous expression assay established for assessing photoreceptor action spectra (36), *Platynereis* r-Opsin1 exhibits an irradiance response peak in the blue range (λ_max_= approximately 470 nm) (37), similar to the peak of its human melanopsin homolog. When we assessed the respective sensitivities of both receptors in side-by-side comparisons, the half-maximal effective irradiation (EI_50_) of *Platynereis* r-Opsin1 (2.3 × 10^10^ photons cm^−2^ s^−^1) was ∼100 times lower than that of melanopsin (2.5 × 10^12^ photons cm^−2^ s^−1^; Fig. 6*A*), indicating a remarkably high sensitivity of *Pdu-*r-Opsin1.

In the animal, this molecular sensitivity is combined with a high abundance of r-Opsin1: on the transcript level, a cellular profiling analysis revealed that *r-opsin1* is one of the topmost expressed genes in *Platynereis* adult eye photoreceptors, outnumbering a distinct coexpressed opsin—*r-opsin3*—by nearly three orders of magnitude (37). Moreover, in the course of the metamorphic changes that occur during the days immediately prior to swarming, the outer segments of the eye photoreceptors—where Opsin molecules are concentrated in tightly packed membrane stacks—extend to around twice their length, suggesting an even increased sensitivity (38). All these facts imply that r-Opsin1 acts as a particularly high-sensitive light detector at the time of swarming.

To test whether r-Opsin1 was indeed required to mediate the impact of moonlight on the timing of swarming onset, we capitalized on an existing *r-opsin1^−17/-17^* loss-of-function allele (37). Under a constant darkness (DD) regime, *r-opsin1^–/–^* mutants were indistinguishable from wild type (*SI Appendix*, Fig. S7*A*), while they maintained synchronization under constant naturalistic sunlight, by this differing from wild type (*SI Appendix*, Fig. S7*B*). They also differ from *l-cry^–/–^* mutants in that they advance their timing under LL (*SI Appendix*, Fig. S7*B* vs. Fig. 2*C*). As discussed in *SI Appendix*, *SI Text*, both the maintained synchronization and clock advancement might be connected to mechanisms underlying photoperiodic adjustments of the PCC clock, which also exist (*SI Appendix*, Fig. S3*B*).

When we exposed *r-opsin1^–/–^* mutants to the naturalistic FM regime they failed to reset swarming time to earlier hours (Fig. 6*B*) but remained restricted to nocturnal hours. This contrasts with *l-cry^−/−^* mutants under the same paradigm (Fig. 6*C*). Our data are consistent with the notion that r-Opsin1 is critical for moonlight detection but not sunlight versus moonlight discrimination. In order to further test for r*-*Opsin1’s requirement for moonlight detection, we subjected homozygous *r-opsin1^17/-17^* mutants and related wild-type individuals for 5 d to the established waning moon paradigm (Fig. 1*E*), providing naturalistic moonlight during the second half of the night (Fig. 6*D*). *r-opsin1^−/−^* animals exhibited a significantly reduced ability to shift their swarming onset to the dark portion of the night compared to wild types (Fig. 6*D*). This difference became even stronger with naturalistic moonlight at lower intensities (as this would be the case for the natural waning moon) (Fig. 6*E*). Finally, we wondered if *r-opsin1^–/–^* mutants would also exhibit a reduced ability to reset the PCC clock under constant moonlight. Under constant moonlight at naturalistic FM (Fig. 6*F*) or waning moon (Fig. 6*G*) light intensities, *r-opsin1^–/–^* mutants were indistinguishable from wild type.

Taken together with the results under the more naturalistic mixed moonlight/sunlight regimes, this finding let us conclude that r-Opsin1 specifically enables the worms to detect moonrise to align the PCC clock accordingly. Under constant moonlight, however, the continuous activation of L-Cry appears to be sufficient for PCC clock period shortening. At present, we cannot fully explain the difference in *r-opsin1* requirement for LM and MM regimes. Possibly, the LM regime does not provide a sufficiently long moonlight illumination for L-Cry to be fully activated, and/or the ability of L-Cry to advance the clock is restricted to certain phases of the clock. Future work will be required to understand the detailed mechanisms of how naturalistic light impacts on the oscillator under these conditions.

**Fig. 7.**
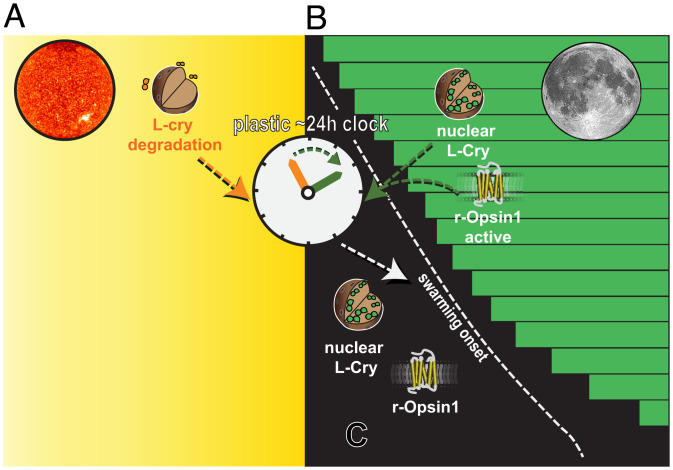
Model of how the combinatorial responses of L-Cry and r-Opsin1 might encode sunlight, moonlight and darkness to adjust the plastic circadian/circalunidian clock to control the hour of swarming onset. (*A*) Sunlight fully photoreduces L-Cry (1) and triggers its degradation (Fig. 3 *B* and *C*) (1) to likely synchronize the PCC clock to the 24-h solar day. (*B*) Prolonged moonlight likely activates nuclear L-Cry in a noncanonical fashion, as L-Cry is required to shorten the PCC period length under prolonged moonlight conditions (Fig. 2*B*). r-Opsin1 with its rapid activation and high light sensitivity (Fig. 6*A*) is critical to correctly adjust the PCC clock to the dim light of the waning moon (Fig. 6 *D* and *E*) in order to optimize swarming onset time prior to naturally occurring moonrise. (*C*) Under darkness, L-Cry is abundantly present in the nucleus, but neither r-opsin1 or L-Cry are photoactivated.

The distinct phenotypes of the *l-cry^–/–^* versus *r-opsin1^–/–^* mutants argue for distinct roles of L-Cry and r-Opsin1 in decoding naturalistic moonlight and adjusting the PCC clock (Fig. 7): we hypothesize that the different subcellular levels of L-Cry during night and day (Fig. 7 *A*–*C*) provide key information on the level of irradiance. For the organism, this would harbor information on light valence, as the sources of light—sun versus moon—have significantly different temporal/ecological meanings.

Whereas *r-opsin1* has no involvement in the entrainment of the monthly oscillator (1), our data indicate that in case of the PCC clock adjustment, it is relevant for mediating acute dim light information. The combination of L-Cry’s and r-Opsin1’s properties therefore allow the PCC clock to distinguish between not only sunlight, FM, and new moon but also the progressive phases of the waning moon, which are particularly relevant to set the right swarming hour (Fig. 7).

## Discussion

Here we uncover an ∼24-h endogenous oscillator in marine broadcast-spawning worms that exhibits marked, moonlight-dependent plasticity in its period length upon extended moonlight illumination, as it occurs during FM periods. The clock is also adjusted by moonrises during the waning moon phase. It is currently unclear if the latter adjustment also relies on a change in period length or by phase resetting. Furthermore, during nights when swarming time is fine-tuned by the light of the moon, the PCC is not reset by the following solar light (sunrise).

The modulation of the worms’ ∼24-h oscillator by naturalistic moonlight provides a plausible model for how worms synchronize their nuptial dance, targeting a specific hour during the dark portion of moonlit nights. Restricting swarming behavior to the dark portion of the night might be advantageous to avoid predators that hunt during moonlight. On a mechanistic level, we suggest that this PCC clock shares elements with the conventional core circadian oscillator and reveal two highly sensitive light receptors, r-Opsin1 and L-Cry, that are critical to sense and interpret naturalistic moonlight.

Sensitivity to moonlight is directly relevant for a broad panel of marine broadcast spawners. The challenge of tagging nocturnal light information with the correct valence, however, likely extends beyond this specific ecological context. The classical categorization of organisms into nocturnal versus diurnal species (39, 40) typically neglects the aspect of moonlight. Any animal entraining its ∼24-h clock to light will need to correctly interpret the occurrence of nocturnal light. Even though it has been shown that the circadian system of many species is sensitive to light levels as low as moonlight intensity, such as in flies (19, 20) and mice (41), chronobiological studies have so far spent relatively little effort in dissecting how animal clocks prevent potential disturbance by moonlight and interpret naturalistic light regimes that combine both sunlight and moonlight.

The data presented here provide possible mechanistic explanations for the ability of the PCC clock to decode a combined sunlight and moonlight regime. A first tier is connected to the specific properties of cryptochrome: whereas under naturalistic moonlight, *Platynereis* L-Cry protein levels remain elevated, comparable to dark conditions, and are predominantly localized to the nucleus, the onset of sunlight causes a rapid degradation, with residual L-Cry protein found in the cytoplasm. On the biochemical level, L-Cry is highly sensitive to naturalistic moonlight. Moonlight evokes a different state in L-Cry than sunlight (see extensive comparison of sunlight vs. moonlight in ref. 1). Based on these detailed biochemical studies, including multiangle light scattering and size exclusion chromatography analyses, we hypothesize that *Pdu-*L-Cry functions as a dimer with different light sensitivities of each of the two monomers, potentially arising from different quantum yields for FAD photoreduction (1). This would allow for the differential response to high-intensity sunlight versus very low intensity moonlight, as moonlight can only photoreduce the flavin cofactor in the low-light sensitive L-Cry monomer, whereas sunlight can photoreduce both (1). Dimers as part of the photoresponse mechanism have been well documented for Cryptochromes in plants. These Cryptochromes form dimers or even multimers upon illumination, which are essential for the light response (42–44). Even though plant Cry2 members are frequently called low-light receptors, their responses have only been analyzed under light conditions that still mimic sunlight (42, 44), which are magnitudes away from moonlight (*SI Appendix*, Fig. S2, and ref. 45). Thus, the mechanism by which *Pdu-*L-Cry responds to the very dim moonlight has to be more efficient even compared to the low-light sensors of the plant Cry2 family, while L-Cry also still retains the ability to detect high-intensity sunlight. The dimer model of L-Cry fulfills this requirement (1). In contrast to L-Cry, its direct *Drosophila* ortholog dCRY (14) does not form dimers (31). This difference is consistent with the lack of photoreduction of purified dCRY under naturalistic moonlight that we report here.

Taken together, our data are consistent with the idea that—besides the canonical strong-light induced degradation-based signaling pathway for cytoplasmic Cryptochrome—L-Cry, but not dCRY, possesses a second, dim-light–induced, nuclear mode of signaling. Its presence in the nucleus and absence of degradation under moonlight makes it tempting to speculate that L-Cry could function as a transcriptional repressor. Notably, *Drosophila* Cryptochrome has been proposed to function as transcriptional repressor in the flies’ peripheral tissues under darkness (46). However, whereas the worms’ vertebrate/animalCry2 ortholog tr-Cry (14) showed transcriptional repressor activity in a heterologous S2 cell assay, *Pdu-*L-Cry did not under the same conditions (5). Future work may determine if (worm) cell type–specific factors could be required for L-Cry to exert transcriptional activity or if it could rather regulate posttranscriptional responses.

A second lead on how moonlight can impact on ∼24-h timing is provided by our identification of r-Opsin1 as a second moonlight sensor. Whereas it remains to be uncovered how the r-Opsin1–dependent signals tie in with the different signaling states of L-Cry, the existence of two distinct sensors already opens up the possibility for a combinatorial setup, in which the (nuclear) presence of L-Cry allows an Opsin-dependent signal to be interpreted as moonlight, whereas the reduction of L-Cry upon sunlight illumination allows an incoming Opsin-dependent signal to be interpreted as sunlight (Fig. 7). Just the combination of the two signals alone would be sufficient for the light distinction. This combinatorial model could also explain the situation of *Drosophila*, where purified dCRY biochemically does not respond to naturalistic moonlight but is genetically required to shield the *Drosophila* circadian clock from moonlight impact. Opsin-based light input also plays a role for circadian entrainment, including the detection of light at moonlight intensity (47), and together with the information from dCRY, the natural source of light could be discriminated. For a diurnal/crepuscular species such as *D. melanogaster*, the main importance of the discrimination between sunlight and moonlight is to reduce the impact of moonlight on its ∼24-h clock and not to tune it to moonlight. Therefore, the moonlight sensitivity of the cryptochrome component itself is a dispensable feature for the fruitfly’s dCRY. In contrast, the nocturnal bristle worm fine-tunes its plastic PCC with moonlight. A direct sensitivity of L-Cry to moonlight is likely beneficial for this moonlight-responding system and hence exists biochemically for L-Cry.

Evidence for plasticity of the conventional circadian clock has started to emerge from other marine systems: work on the circatidal oscillators of oysters maintained under controlled laboratory conditions revealed that core circadian clock genes exhibit ∼12.4-h cycles under constant darkness, whereas the transcripts of the same genes cycle with an ∼24-h oscillation under light/dark conditions (48). This provides evidence for the ability of the canonical clock to alternate between circadian (∼24 h) and (semi)circalunidian (∼12.4 h/∼24.8 h) periodicities. Of note, switches between circadian and circalunidian cycles might also occur in humans. For instance, mood switches of bipolar patients correlate with a period lengthening of their body temperature cycles that looks as if the circadian timing system can be intermittently entrained to a 24.8-h rhythm (49). Moreover, already classical chronobiological studies documented changes of the ∼24-h clock periodicity under dim light in various organisms, including birds, mice, hamsters, and humans (50, 51), as well as the fruit fly *D. melanogaster* (22). Whereas the meaning of these results had remained enigmatic, they could well be explained by the conceptual framework of combined solar and lunar light cues that we present in our study. We anticipate that research on organisms for which lunar impact is of known biological relevance will be key to disentangle the interplay of solar and lunar timing cues.

## Materials and Methods

Detailed methods on the following subjects are available in *SI Appendix*, *SI Methods*: natural light measurements, behavioral setup and analyses of swarming onset in worms, Western blots, immunohistochemistry, Period oscillations in *Drosophila* clock neurons, spectral sensitivity comparison of opsins, casein kinase inhibitor treatment and qPCR analyses, recombinant expression and purification of L-Cry and dCRY proteins, and UV/VIS spectroscopy of L-Cry and dCRY.

### Worm Culture.

Worms were grown as described previously (10). In short, worms were kept in plastic boxes filled with a 1:1 mixture of natural sea water and artificial sea water (30% Tropic Marine) and exposed to a 16:8 h light:dark light regime. To entrain their circalunar clock, worms received eight nights of continuous nocturnal light each month to mimic FM.

#### Strains.

##### l-cry^−/−^.

Homozygous *lcry^Δ34/Δ34^* worms were obtained by crosses between *lcry^Δ34/^^+^* individuals generated and maintained in the VIO-strain background (see ref. 1). Wild-type worms used in the experiments for comparison to *l-cry^−/−^* worms were derived from the respective *lcry^+/+^* relatives obtained in the crosses.

##### r-opsin1^−/−^.

Homozygous *r-opsin^Δ17^*^*/Δ17*^ worms were obtained by crosses between *r-opsin^Δ17^^/+^* individuals generated in the pMos{rops::egfp}^vbci2^ transgenic strain (37). Wild-type worms used for comparisons were derived from the pMos{rops::egfp}^vbci2^ transgenic strain in which the mutant was generated.

### Recording of Locomotor Activity in *D. melanogaster*.

Locomotor activity was recorded under constant temperature (20 °C) from 0- to 1-d-old male Canton-S and *cry^01^* (Canton-S background) flies using the *Drosophila* Activity Monitors from Trikinetics Incorporation (28). Flies were recorded first for 5 d under 12 h light–12 h dark cycles (LD with ∼100 lx standard white light LED) and then for 7 d under 12 h light–12 h artificial moonlight cycles (LM cycles; for spectrum and intensity of artificial moonlight, see *SI Appendix*, Fig. S2*C*). The average actograms and the centers of maximal activity were calculated and plotted with ActogramJ (52). The phases of evening activity maxima under LM conditions were determined using the ActogramJ tool “acrophase.” To test for differences in the acrophase of wild-type and *cry^01^* flies at LM4, an unpaired Student test was performed.

### Imaging and Quantification of L-Cry Staining.

Imaging of the worm heads was done on a Zeiss laser scanning confocal microscope (model LSM 700), using Plan-Apochromat 25X and Plan-Apochromat 40X objectives, a transmission photomultiplier tube (T-PMT) detection system and Zeiss ZEN 2012 software. Lasers used were at 405 nm and 555 nm. Image analysis was performed using the software Fiji/ImageJ (53).

Nuclei were segmented using Cellpose (54) on the DAPI channel images. Subsequently, the segmented nuclei were used as regions of interest (ROIs) in Fiji/ImageJ (53) to quantify the signal intensity by calculating the corrected total cell fluorescence (CTCF) as follows: CTCF = Area_ROI1_ * Mean_ROI1_ − Area_ROI1_ * Mean_backgroundROIs_. CTCF was determined for the entire brain area as well and used for calculation of the signal intensity of nonnuclei, which was considered  cytoplasmic (CTCF_cytoplasm_ = CTCF_total_ − CTCF_nuclei_).

### Statistical Analyses.

We used one-way ANOVA followed by Dunnett’s test to test if the timing of swarming onset during LD conditions differs compared to conditions where worms are subjected to moonlight conditions on top of an LD cycle. We used two-way ANOVA followed by Sidak’s test to test if and during which days the timing of swarming onset differs between mutant and wild types across different days of a behavioral experiment. To compare if two sets of data had different variances, an F test as part of *t* test statistics was performed. Swarming onset data are shown as individual data points and additionally represented as box plots with whiskers reaching to the maximal and minimal values.

Western blot data, which were used to assess head L-Cry levels during sunlight, moonlight, and darkness conditions, were analyzed with one-way ANOVA followed by Tukey’s multiple comparison test to test for significant differences in L-Cry abundance between the different light conditions.

To compare period oscillation in the different cell groups between *cry01* mutants and wild-type flies over different ZTs we used two-way ANOVA followed by Sidak’s test.

## Supplementary Material

Supplementary File

Supplementary File

## Data Availability

Light measurement data and data underlying the analyses of swarming times have been deposited in the Dryad Digital Repository (https://datadryad.org) (DOI: 10.5061/dryad.2v6wwpzkr) (55). All other data are included in the manuscript and supporting information. Genetic animal strains used in the described work will be shared upon request with qualified researchers for their own use.

## References

[1] B. Poehn , A Cryptochrome adopts distinct moon- and sunlight states and functions as moonlight interpreter in monthly oscillator entrainment. bioRxiv [Preprint] (2021). 10.1101/2021.04.16.439809. Accessed 23 February 2022. PMC9445029 36064778

[2] J. Zantke, S. Bannister, V. B. V. Rajan, F. Raible, K. Tessmar-Raible, Genetic and genomic tools for the marine annelid *Platynereis dumerilii*. Genetics 197, 19–31 (2014). 24807110 10.1534/genetics.112.148254 PMC4012478

[3] S. Ranzi, Ricerche sulla biologia sessuale degli Annelidi. Pubbl. Stn. Zool. Napoli 11, 271–292 (1931).

[4] C. Hauenschild, Lunar periodicity. Cold Spring Harb. Symp. Quant. Biol. 25, 491–497 (1960). 13712278 10.1101/sqb.1960.025.01.051

[5] J. Zantke , Circadian and circalunar clock interactions in a marine annelid. Cell Rep. 5, 99–113 (2013). 24075994 10.1016/j.celrep.2013.08.031 PMC3913041

[6] H. Caspers, Spawning periodicity and habitat of the palolo worm *Eunice viridis* (Polychaeta: Eunicidae) in the Samoan Islands. Mar. Biol. 79, 229–236 (1984).

[7] G. R. Gaston, J. Hall, Lunar periodicity and bioluminescence of swarming *Odontosyllis luminosa* (Polychaeta: Syllidae) in Belize. Gulf Caribb. Res. 12, 47–51 (2000).

[8] N. Kronfeld-Schor , Chronobiology by moonlight. Proc. Biol. Sci. 280, 20123088 (2013). 23825199 10.1098/rspb.2012.3088 PMC3712431

[9] G. Andreatta, K. Tessmar-Raible, The still dark side of the moon: Molecular mechanisms of lunar-controlled rhythms and clocks. J. Mol. Biol. 432, 3525–3546 (2020). 32198116 10.1016/j.jmb.2020.03.009 PMC7322537

[10] S. Schenk , Combined transcriptome and proteome profiling reveals specific molecular brain signatures for sex, maturation and circalunar clock phase. eLife 8, 1–39 (2019). 10.7554/eLife.41556 PMC6377233 30767890

[11] V. B. Veedin Rajan , Seasonal variation in UVA light drives hormonal and behavioural changes in a marine annelid via a ciliary opsin. Nat. Ecol. Evol. 5, 204–218 (2021). 33432133 10.1038/s41559-020-01356-1 PMC7611595

[12] M. van der Steen, Spectrum of moon light. www.olino.org/blog/us/articles/2015/10/05/spectrum-of-moon-light. Accessed 8 October 2020.

[13] O. Levy , Light-responsive cryptochromes from a simple multicellular animal, the coral *Acropora millepora*. Science 318, 467–470 (2007). 17947585 10.1126/science.1145432

[14] P. Oliveri , The cryptochrome/photolyase family in aquatic organisms. Mar. Genomics 14, 23–37 (2014). 24568948 10.1016/j.margen.2014.02.001

[15] L. E. Foley, P. Emery, *Drosophila* cryptochrome: Variations in blue. J. Biol. Rhythms 35, 16–27 (2020). 31599203 10.1177/0748730419878290 PMC7328257

[16] L. Zhang , Dissociation of circadian and circatidal timekeeping in the marine crustacean *Eurydice pulchra*. Curr. Biol. 23, 1863–1873 (2013). 24076244 10.1016/j.cub.2013.08.038 PMC3793863

[17] S. Smadja Storz , Casein kinase 1δ activity: A key element in the zebrafish circadian timing system. PLoS One 8, e54189 (2013). 23349822 10.1371/journal.pone.0054189 PMC3549995

[18] M. Oren , Profiling molecular and behavioral circadian rhythms in the non-symbiotic sea anemone *Nematostella vectensis*. Sci. Rep. 5, 11418 (2015). 26081482 10.1038/srep11418 PMC4476465

[19] A. Klarsfeld , Novel features of cryptochrome-mediated photoreception in the brain circadian clock of *Drosophila*. J. Neurosci. 24, 1468–1477 (2004). 14960620 10.1523/JNEUROSCI.3661-03.2004 PMC6730330

[20] J. Hirsh , Roles of dopamine in circadian rhythmicity and extreme light sensitivity of circadian entrainment. Curr. Biol. 20, 209–214 (2010). 20096587 10.1016/j.cub.2009.11.037 PMC2811851

[21] T. Yoshii , *Drosophila cry^b^* mutation reveals two circadian clocks that drive locomotor rhythm and have different responsiveness to light. J. Insect Physiol. 50, 479–488 (2004). 15183277 10.1016/j.jinsphys.2004.02.011

[22] R. J. Konopka, C. Pittendrigh, D. Orr, Reciprocal behaviour associated with altered homeostasis and photosensitivity of *Drosophila* clock mutants. J. Neurogenet. 6, 1–10 (1989). 2506319 10.3109/01677068909107096

[23] L. Kempinger, R. Dittmann, D. Rieger, C. Helfrich-Förster, The nocturnal activity of fruit flies exposed to artificial moonlight is partly caused by direct light effects on the activity level that bypass the endogenous clock. Chronobiol. Int. 26, 151–166 (2009). 19212834 10.1080/07420520902747124

[24] W. Bachleitner, L. Kempinger, C. Wülbeck, D. Rieger, C. Helfrich-Förster, Moonlight shifts the endogenous clock of *Drosophila melanogaster*. Proc. Natl. Acad. Sci. U.S.A. 104, 3538–3543 (2007). 17307880 10.1073/pnas.0606870104 PMC1805525

[25] S. Vanin , Unexpected features of *Drosophila* circadian behavioural rhythms under natural conditions. Nature 484, 371–375 (2012). 22495312 10.1038/nature10991

[26] M. Schlichting, R. Grebler, P. Menegazzi, C. Helfrich-Förster, Twilight dominates over moonlight in adjusting *Drosophila*’s activity pattern. J. Biol. Rhythms 30, 117–128 (2015). 25838418 10.1177/0748730415575245

[27] E. Dolezelova, D. Dolezel, J. C. Hall, Rhythm defects caused by newly engineered null mutations in *Drosophila*’s *cryptochrome* gene. Genetics 177, 329–345 (2007). 17720919 10.1534/genetics.107.076513 PMC2013679

[28] M. Schlichting, C. Helfrich-Förster, Photic entrainment in *Drosophila* assessed by locomotor activity recordings. Methods Enzymol. 552, 105–123 (2015). 25707274 10.1016/bs.mie.2014.10.017

[29] C. Kistenpfennig , A tug-of-war between cryptochrome and the visual system allows the adaptation of evening activity to long photoperiods in *Drosophila* *melanogaster*. J. Biol. Rhythms 33, 24–34 (2018). 29179610 10.1177/0748730417738612

[30] A. Chatterjee , Reconfiguration of a multi-oscillator network by light in the *Drosophila* circadian clock. Curr. Biol. 28, 2007–2017.e4 (2018). 29910074 10.1016/j.cub.2018.04.064 PMC6039274

[31] A. Berndt , A novel photoreaction mechanism for the circadian blue light photoreceptor *Drosophila* cryptochrome. J. Biol. Chem. 282, 13011–13021 (2007). 17298948 10.1074/jbc.M608872200

[32] N. Hoang , Human and *Drosophila* cryptochromes are light activated by flavin photoreduction in living cells. PLoS Biol. 6, e160 (2008). 18597555 10.1371/journal.pbio.0060160 PMC2443192

[33] D. Arendt, K. Tessmar, M.-I. M. de Campos-Baptista, A. Dorresteijn, J. Wittbrodt, Development of pigment-cup eyes in the polychaete *Platynereis dumerilii* and evolutionary conservation of larval eyes in Bilateria. Development 129, 1143–1154 (2002). 11874910 10.1242/dev.129.5.1143

[34] N. Randel, L. A. Bezares-Calderón, M. Gühmann, R. Shahidi, G. Jékely, Expression dynamics and protein localization of rhabdomeric opsins in *Platynereis* larvae. Integr. Comp. Biol. 53, 7–16 (2013). 23667045 10.1093/icb/ict046 PMC3687135

[35] B. Backfisch , Stable transgenesis in the marine annelid *Platynereis dumerilii* sheds new light on photoreceptor evolution. Proc. Natl. Acad. Sci. U.S.A. 110, 193–198 (2013). 23284166 10.1073/pnas.1209657109 PMC3538230

[36] H. J. Bailes, R. J. Lucas, Human melanopsin forms a pigment maximally sensitive to blue light (λ_max_ ≈ 479 nm) supporting activation of G_q/11_ and G_i/o_ signalling cascades. Proc. R. Soc. B. 280, 20122987 (2013). 10.1098/rspb.2012.2987 PMC3619500 23554393

[37] R. Revilla-I-Domingo , Characterization of cephalic and non-cephalic sensory cell types provides insight into joint photo- and mechanoreceptor evolution. eLife 10, 1–31 (2021). 10.7554/eLife.66144 PMC8367381 34350831

[38] A. Fischer, J. Brökelmann, Das Auge von *Platynereis dumerilii* (Polychaeta). Z Zellforsch. Mikrosk. Anat. 71, 217–244 (1966). 4864472

[39] C. S. Pittendrigh, Circadian rhythms and the circadian organization of living systems. Cold Spring Harb. Symp. Quant. Biol. 25, 159–184 (1960). 13736116 10.1101/sqb.1960.025.01.015

[40] J. Aschoff, Circadian rhythms: Influences of internal and external factors on the period measured in constant conditions. Z. Tierpsychol. 49, 225–249 (1979). 386643 10.1111/j.1439-0310.1979.tb00290.x

[41] C. M. Altimus , Rod photoreceptors drive circadian photoentrainment across a wide range of light intensities. Nat. Neurosci. 13, 1107–1112 (2010). 20711184 10.1038/nn.2617 PMC2928860

[42] C. Lin , Enhancement of blue-light sensitivity of *Arabidopsis* seedlings by a blue light receptor cryptochrome 2. Proc. Natl. Acad. Sci. U.S.A. 95, 2686–2690 (1998). 9482948 10.1073/pnas.95.5.2686 PMC19462

[43] Y. Sang , N-terminal domain-mediated homodimerization is required for photoreceptor activity of *Arabidopsis* CRYPTOCHROME 1. Plant Cell 17, 1569–1584 (2005). 15805487 10.1105/tpc.104.029645 PMC1091775

[44] Q. Wang , Photoactivation and inactivation of *Arabidopsis* cryptochrome 2. Science 354, 343–347 (2016). 27846570 10.1126/science.aaf9030 PMC6180212

[45] M. P. S. Dekens, N. S. Foulkes, K. Tessmar-Raible, Instrument design and protocol for the study of light controlled processes in aquatic organisms, and its application to examine the effect of infrared light on zebrafish. PLoS One 12, e0172038 (2017). 28212399 10.1371/journal.pone.0172038 PMC5315407

[46] B. Collins, E. O. Mazzoni, R. Stanewsky, J. Blau, *Drosophila* CRYPTOCHROME is a circadian transcriptional repressor. Curr. Biol. 16, 441–449 (2006). 16527739 10.1016/j.cub.2006.01.034

[47] C. Helfrich-Förster, Light input pathways to the circadian clock of insects with an emphasis on the fruit fly *Drosophila melanogaster*. J. Comp. Physiol. A Neuroethol. Sens. Neural Behav. Physiol. 206, 259–272 (2020). 31691095 10.1007/s00359-019-01379-5 PMC7069913

[48] D. Tran, M. Perrigault, P. Ciret, L. Payton, Bivalve mollusc circadian clock genes can run at tidal frequency. Proc. Biol. Sci. 287, 20192440 (2020). 31910786 10.1098/rspb.2019.2440 PMC7003461

[49] T. A. Wehr, Bipolar mood cycles and lunar tidal cycles. Mol. Psychiatry 23, 923–931 (2018). 28115741 10.1038/mp.2016.263 PMC5524624

[50] J. Aschoff, Exogenous and endogenous components in circadian rhythms. Cold Spring Harb. Symp. Quant. Biol. 25, 11–28 (1960). 13684695 10.1101/sqb.1960.025.01.004

[51] J. Aschoff, Circadian rhythms in man. Science 148, 1427–1432 (1965). 14294139 10.1126/science.148.3676.1427

[52] B. Schmid, C. Helfrich-Förster, T. Yoshii, A new ImageJ plug-in “ActogramJ” for chronobiological analyses. J. Biol. Rhythms 26, 464–467 (2011). 21921300 10.1177/0748730411414264

[53] J. Schindelin , Fiji: An open-source platform for biological-image analysis. Nat. Methods 9, 676–682 (2012). 22743772 10.1038/nmeth.2019 PMC3855844

[54] C. Stringer, T. Wang, M. Michaelos, M. Pachitariu, Cellpose: A generalist algorithm for cellular segmentation. Nat. Methods 18, 100–106 (2021). 33318659 10.1038/s41592-020-01018-x

[55] M. Zurl ., Data for paper: Two light sensors decode moonlight versus sunlight to adjust a plastic circadian/circalunidian clock to moon phase. Dryad Digital Repository (2022). 10.5061/dryad.2v6wwpzkr. Deposited 23 May 2022.

