# Supplementary material for: Two light sensors decode moonlight versus sunlight to adjust a plastic circadian/circalunidian clock to moon phase
Source: Proc Natl Acad Sci U S A. 2022 May 27;119(22):e2115725119. doi: 10.1073/pnas.2115725119 (PMC9295771; doi:10.1073/pnas.2115725119)
Supplement: Supplementary File [file pnas.2115725119.sapp.pdf]

PNAS

Supplementary Information for

Two light sensors decode moonlight versus sunlight to adjust a plastic circadian/circalunidian clock to moon phase

Martin Zurl, Birgit Poehn, Dirk Rieger, Shruthi Krishnan, Dunja Rokvic, Vinoth Babu Veedin Rajan, Elliot Gerrard, Matthias Schlichting, Lukas Orel, Aida Ćorić, Robert J. Lucas, Eva Wolf, Charlotte Helfrich-Förster, Florian Raible and Kristin Tessmar-Raible

Correspondence:

Florian Raible: florian.raible@univie.ac.at

Kristin Tessmar-Raible: kristin.tessmar@maxperutzlabs.ac.at

This PDF file includes:

Supplementary Text

Supplementary Methods

Figures S1 to S7

Legend for Movie S1

Other supplementary materials for this manuscript include the following:

Movie S1

Supplementary Information Text

As shown in **Fig.S7B**, *r-opsin1* mutant worms exhibit less desynchronization of swarming times under constant naturalistic sunlight (LL) when compared to wild-type controls. One possible explanation for this phenomenon might come from the comparison to the mammalian SCN and its responses to constant (relatively strong) light. As we have shown before, the posterior adult worm brain harbors two symmetrically localized oval-shaped domains that each consist of >100 cells, and that are characterized by a high level of core circadian clock gene expression (1), a setup that differs from the few circadian clock cells in the fruitfly model. We have furthermore shown that the head harbors a central circadian oscillator (2). It is thus a plausible possibility that in analogy to the mammalian SCN, the *Platynereis* clock cells also form an interconnected network, which drives the circadian behavioral rhythms of the worm.

In mammals, it has been shown that constant light leads to a desynchronization of these cells relative to each other (and between the two SCNs), and that this correlates with the degree of arrhythmic behavior (3). It is thought that under natural light conditions this plasticity is used by the animals to acutely adjust their circadian timing to changes in photoperiod, mediated by the peptide VIP and ArgVasopression (4). At least for VIP it has been shown that its effects are dose dependent: Concentrations below 100nM synchronize the SCN cells, while concentrations above this level desynchronize the same system (5). Naturally, VIP in the SCN neurons is controlled by light input, which is relayed by melanopsin-positive retinal ganglion cells, and is important for the light resetting of daily rhythms, as it would naturally occur during photoperiod changes (6).

Platynereis r-Opsin1 and r-Opsin3 are direct orthologs of mammalian melanopsin and co-express in the worms' eye rhabdomeric photoreceptor cells, with *r-opsin1* expression being almost 10^3-fold higher than *r-opsin3* (7). The photoreceptors might be considered the worms' evolutionary cell type correlate to the mammalian melanopsin+ retinal ganglion cells, based on several layers of evidence, including the expression of several marker genes and projections into the posterior brain nuclei that harbor prominent circadian clock gene expression (8, 9). Furthermore, a common orthologous receptor for PACAP/VIP/PTH/GCG signaling is very ancient (10) and in place in *Platynereis* (11). The same holds for a direct ortholog of ArgVasopressin and its receptors (11–13).

It is thus conceivable that systems similar to the mammalian light-regulated PACAP/VIP and ArgVasopressin systems function in the posterior oval-shaped timing domain of *Platynereis* to synchronize/desynchronize the individual ~24hr oscillatory neurons in response to r-Opsin-mediated daylight input to adjust the system to photoperiod changes. In case of very strong light – as in our naturalistic sunlight LL regime – this would result in the observed desynchronization of the wild-type swarming behavior. By contrast, in the r-Opsin1-/- mutants, a light signal would still be perceived by the remaining r-Opsin3, but strongly reduced, as would any potentially coupled PACAP/VIP/PTH/GCG signaling. In analogy to the outlined dependency of cellular desynchronization/synchronization in the SCN on VIP concentration, such a scenario could result in the observed retention of swarming synchrony of *r-opsin1-/-* worms (**Fig. S7B**).

In addition to the maintained swarming synchrony of *r-opsin1-/-* animals under constant naturalistic sunlight, it can be noted that these mutants exhibit a <24hr period length. As mentioned above, the phenotypes of the mammalian SCN neuronal network under the LL- paradigm are thought to reflect a plastic system, that is under natural conditions used to adjust to photoperiod changes (4). Thus, the mentioned evolutionary conservation and our results that extended photoperiod also advances the worms' ~24hr timing (**Fig. S3B**), are highly consistent with the interpretation that the timing advancement visible in the synchronized *r-opsin1-/-* animals might reflect a photoperiod response.

Supplementary Methods.

Natural light measurements. Under-water measurements of natural sun- and moonlight at the habitat of *Platynereis* were acquired using a RAMSES-ACC-VIS hyperspectral radiometer (TriOS GmbH) for UV to IR spectral range (see ref. (13) for details). Radiometers were placed at 4m and 5m water depth close to *Posidonia oceanica* meadows, which are a natural habitat for *P. dumerilii*. Measurements were recorded automatically every 15min across several weeks in the winter 2011/2012 (at 5m depth) and during spring 2011 (at a 4m depth). To obtain an exemplary sunlight spectrum, the sunlight measurements taken at 5m depth between 10 am-4 pm on Nov. 25, 2011 were averaged. To obtain a full moon spectrum for the 5m depth location, measurements taken from 10pm to 1am on a clear full moon night (Nov. 10-11, 2011) were averaged. To control for technical noise caused by the measurement device at these low light intensities, a NM spectrum was obtained by averaging measurements between 7:15pm to 5am on a NM night on Nov 24, 2011, and subtracted from the FM spectrum. The resulting spectrum is plotted in Fig. S2A. To validate that this spectrum is representative of a typical full moon spectrum at the habitat of *Platynereis*, we averaged moonlight measured between 10:15 pm to 2am during a full moon night (April 17-18, 2012) and subtracted a NM spectrum measured two weeks earlier from 4m depth (Fig. S2A). To benchmark these moonlight spectra measured under water with moonlight measured on land, we compared the underwater spectra to a publicly available full moon spectrum measured on land on (Fig. S2A, http://www.olino.org/blog/us/articles/2015/10/05/spectrum-of-moon-light; retrieved on April 14, 2014 in the Netherlands). As expected, light with longer wavelengths was strongly reduced in the underwater measurements compared to the surface spectrum, since light with longer wavelengths penetrates water less efficiently.

Behavioral setup and analyses of swarming onset in worms. All behavioral experiments, except Fig. 1B and Fig. 2F,G were performed with worms that received LD conditions without any nocturnal light (FM) for at least 9 days. Since most *l-cry* mutants spawn during the first 9 nights after the FM stimulus under standard worm culture conditions (14) , the monthly FM stimulus was omitted for *l-cry* mutants and wildtypes in order to test swarming worms without confounding effect of a recent nocturnal (highly artificial) light stimulus on swarming onset.

Sexually maturing worms were placed in seawater filled individual hemispherical concave wells (diameter = 35mm, depth = 15mm) of a custom-made 36-well clear plastic plate. Video recording of worm's behavior over several days was accomplished as described previously (1), using an infrared (λ = 990 nm) LED array (Roschwege GmbH) illuminating the behavioral chamber and an infrared high-pass filter restricting the video camera. Worms were recorded at least until initiation of swarming (Fig. S1A). Naturalistic sun- and moonlight were generated by custom made LEDs (Marine Breeding Systems, St. Gallen, Switzerland) (for spectra and intensities see Fig. S2B,E). Naturalistic sun- and moonlight were

used in all worm experiments, except for data obtained in Figs. 1B, 2F,G, were we used prototype artificial sun- and moonlight LEDs (Fig. S2C).

Spectra were measured with a calibrated ILT950 spectrometer (International Light Technologies Inc., Peabody, USA). To reliable measure the artificial moonlight, the detector was placed 12cm away from the moonlight source, and based on this measurement moonlight intensity was calculated using the inverse square law for worm position, which was ~51 cm away from the moonlight source.

After video recording, an automated tracking software was used to deduce locomotor activity of individual worms across the time of the recording (13). The exported locomotor activity trajectories, which reflect the distance moved of each worm's center point across 6 min time bins, were analyzed in ActogramJ to manually identify the swarming onset moment. In ambiguous cases (e.g. only little movement detected) we manually analyzed the video recordings to identify the moment when a sexually mature worm left its tube, which was regarded as swarming onset. Swarming onset data were plotted and analyzed using GraphPad Prism 8.0 (La Jolla, USA).

ANOVA was used to test if swarming onset was statistically different across the different days of an experiment. This was followed by Dunnetts multiple comparison test, comparing each day of the experiment with swarming onset during LD conditions. To test differences in swarming onset between mutants and wildtypes across different days of an experiment with varying light conditions, 2-way ANOVA was used followed by Sidak´s multiple comparison test. To identify the free-running periodicity under constant light conditions linear regression analysis was performed. The period length was calculated based on the slope of the regression line ± the 95% CI of the slope. Swarming onset data are presented including the individual data points and a box plot. The whiskers of the box blot represent minimal and maximal values.

Western blots. Four anaesthetized worms were decapitated and heads transferred to a 1.5ml tube containing 150 µl RIPA lysis buffer (R0278 Sigma-Aldrich) supplemented with 10% Triton X100 and protease inhibitor (cOmplete Tablets, EDTA-free, EASYpack, Roche) per biological replicate. The tissue was homogenized by grinding using a tightly fitting pestle. All steps on ice. Cell debris was pelleted by centrifugation. Protein concentration of lysates was determined using Bradford reagent (BIORAD). Proteins were separated by SDS-gel electrophoresis (10% Acrylamide) and transferred to nitrocellulose membrane (Amersham™ Protran™ 0,45µm NC, GE Healthcare Lifescience). Quality of transfer was confirmed by staining with Ponceau-S solution (Sigma Aldrich). After 1h of blocking with 5% slim milk powder (Fixmilch Instant, MARESI) in 1xPTW (1xPBS/0.1% TWEEN 20) at room temperature, the membrane was incubated with the appropriate primary antibody, diluted in 2.5% milk/PTW at 4°C O/N. [anti-L-Cry 5E3-3E6-E8 (1:100) and anti-L-Cry 4D4-3E12-E7 (1:100); anti-beta-Actin (Sigma, A-2066, 1: 20.000)]. After 3 rinses with 1xPTW the membrane was incubated with the species specific secondary antibody [anti-Mouse IgG-Peroxidase antibody, (Sigma, A4416, 1:7500); Anti-rabbit IgG-HRP-linked

antibody (Cell Signaling Technology, #7074, 1:7.500] diluted in 1xPTW/1% slim milk powder for 1 hour. After washing, SuperSignal™ West Femto Maximum Sensitivity Substrate kit (Thermo Fisher Scientific) was used for HRP-signal detection and finally signals were visualized by ChemiDoc Imaging System (BIORAD). Bands were quantified in "Image Lab 6.1" (BIORAD)

Immunohistochemistry. Portions of *Platynereis dumerilii* bodies containing head and jaw were dissected and fixed in 4% PFA at 4° C for 24 h. Afterwards, methanol washes at room temperature (r.t., shaking) and a 5-minutes long digestion using Proteinase K (r.t., not shaking) were employed as means of permeabilization. The worm heads and jaws were then post-fixed with 4% PFA for 20 min at r.t. and washed using 1x PTW (PBS-0.1% Tween 20® (Sigma Aldrich)) 5 times for 5 min. This was followed by over-night incubation in a hybridization mixture (15), commonly used for in situ hybridization (at 65° C in water bath; the solution exchanged once, after the first hour of incubation). Several washing steps were performed the following day, at 65° C in a thermo-block, not shaking (washing sequence, solutions and durations: a. 2 times 20 min with 50% formamide/2X standard saline citrate - 0.1% Tween 20® (Sigma Aldrich), SSCT; b. 2 times 10 min with 2X SSCT; c. 2 times 20 min with 0.2X SSCT). Samples were subsequently blocked using 5% sheep serum (Sigma-Aldrich) (r.t., 90 min, shaking) and incubated for at least 36 h (4° C, shaking) in a mixture of two monoclonal antibodies against L-Cry, 5E3-3E6-E8 and 4D4-3E12-E7 (1:100 and 1:50, correspondingly, in 5% sheep serum (Sigma-Aldrich)) (see accompanying manuscript for further details). Next, samples were washed with 1x PTW 3 times for 15 min (r.t., shaking) and a 1 time over night (4° C, shaking). A Cy3 goat anti-mouse IgG secondary antibody (A10521, Thermo Fisher Scientific) was added in dilution 1:400 in 2.5% sheep serum to specifically detect the bound primary antibody (incubation time and conditions, as well as the following washing steps, were the same as those of the primary antibody). To label nuclei, samples were incubated for 30 min in Höchst 33342 (H3570, Thermo Fisher Scientific), diluted 1:2000 (r.t., shaking), washed 3 times for 15 min using 1x PTW and mounted in 87% glycerol (Sigma-Aldrich)/ddH_2O containing 25 mg/ml DABCO (Roth/Lactan). All solutions were made using 1x PTW unless stated otherwise.

Period oscillations in Drosophila clock neurons. To compare the effect of moonlight between *cry* mutants and wildtypes on Period oscillations in the different clock neuron clusters, we entrained 0-1 day old male Canton-S and *cry01* (CantonS background) flies first under 12h light - 12h dark cycles (~100 lx standard white light LED), and then subjected them to artificial moonlight during the night (=LM cycles; for spectrum: Fig. S2D) for another 4 days. At LM4, whole flies were fixed at the indicated ZTs (for 3h) with 4% PFA + 0.1% TritonX100. Flies were then washed for 3x10min in PBT 0.5%, and their brains were dissected. Subsequently, brains were blocked with 5% NGS in PBT 0.5% for 3 hours. Brains were incubated for 48h at 4°C with the following primary antibodies diluted in PBT 0.5% + 5% NGS: rabbit anti-PER (1:1000), mouse anti-Pdf (1:1000). The secondary antibodies were goat anti-rabbit Alexa™ fluor 488 (1:200) and goat anti-mouse Alexa™ 635 (1:200) incubated at 4°C overnight. Before

mounting, brains were washed 6x with PBT 0.5% (last wash with PBT 0.1%) and then mounted in Vectashield H-1000. Images were acquired with TCS SPE Leica confocal microscope using a 20-fold glycerol immersion objective (Leica Mikrosystems, Wetzlar, Germany) and analyzed with ImageJ as described in ref. (16). PER staining intensity in the different pacemaker cell groups was examined in 12-15 brains (one hemisphere per brain) per timepoint and genotype. To obtain PER staining intensity above background for of each cell group, the PER signal of all cells of a cell group in one hemisphere was averaged and background signal measured near this cell group was subtracted. In case not all cells of a specific cell group could be identified, these missing cells were ignored for analysis.

Finally, to obtain an average staining intensity per cell group, the corresponding staining intensities of all 12-15 brain hemispheres sampled during one time points were averaged.

Spectral sensitivity comparison of opsins. To investigate the spectral sensitivity comparison of *Platynereis* r-Opsin1 to human melanopsin, mammalian expression vectors for both opsins were independently co-transfected into HEK293 cells along with an expression vector containing the luminescent calcium sensitive protein, Aeuqorin (pcDNA5/FRT/TO mtAeq) using Lipofectamine 2000 to access the activation of Gαq signaling as shown in previously published work (7, 17). After 6hrs incubation, the medium was changed to DMEM containing 10% FBS and 10uM 9-cis retinal, after which point the cells were protected from light. The following day, medium was changed to L-15 without phenol red, containing 10uM Coelentrazine-h and 10uM 9-cis retinal. Individual wells were briefly exposed to a 2s flash of near monochromatic light (480nm +/- 10nm) produced from an Xenon arc lamp and delivered via a fiber-optic cable fixed ~10cm above the relevant well and accessed for increase in calcium level by measuring the raw luminescence (RLU) signal with a resolution of 0.5s and cycle of 2s. Luminescence was read using a Clariostar (BMG labtech). Light intensity was modified using combinations of 0.9, 0.2 and 0.1 Neutral density filters. RLU measured during dark incubation preceding the light pulse were used as baseline. Maximum response was determined by the peak luminescence value post light flash, normalised to the maximum luminescence value recorded, per opsin, for that experiment. The resultant maximal response value acquired from each replicate were plotted against the irradiance measured for tested wavelength. This irradiance response curve was then fitted with a sigmoidal dose response function to understand the maximum sensitivity of both opsins.

Casein kinase inhibitor treatment and qPCR analyses. Worms were treated with indicated concentrations of PF-670462 for 3 days under LD conditions during new moon. For sampling, worms were first anaesthetized for ca. 10min with a 1:1 mixture of seawater and 7.5% (w/v) MgCl2 solution. The head was then cut behind the posterior eyes with a scalpel at the indicated timepoints. Five heads were pooled per biological replicate, immediately frozen in liquid nitrogen and stored at -80°C until RNA extraction.

For RNA extraction, 350µl of RNAzol RT (Sigma-Aldrich) were added to the samples and lysis was performed with TissueLyser II (Qiagen) at 30Hz for 2min. Afterwards, RNA was extracted using Direct-zol RNA Miniprep kit (Zymo Research) following the manufacturer's instructions with additional on-column DNaseI digest. RNA was eluted in 34µl of nuclease-free water.

Total RNA (300ng per sample) was reverse transcribed using QuantiTect Reverse Transcription Kit (Qiagen). The resulting cDNA was diluted to a volume of 60µl. qPCR reactions were performed in 20µl total volume with Luna Universal qPCR Master Mix (New England Biolabs). Target genes and reference controls were analysed in duplicate reactions for all samples. Plate control cDNA and -RT controls were included on each plate. cdc5 was used as reference gene (1). Expression levels were calculated using the Δct method. Relative expression values were calculated with the formula: relative expression = 2 -Δct.

Recombinant expression and purification of L-Cry and dCRY proteins. L-Cry was expressed and purified from insect cells as described in (14). N-terminally His6-tagged dCRY was expressed in *Spodoptera frugiperda* (*Sf9*) insect cells using a pFastBac HTb expression vector (18). 1 L of 1 * 10^6 *Sf9* cells/ml in sf900II media were transfected with P1 virus stock and incubated at 27°C for 72 h. Harvested cell pellets were resuspended in lysis buffer (25 mM Tris pH 8.0, 300 mM NaCl, 20 mM imidazole, 5% glycerol, 5 mM β-mercaptoethanol) and lysed by sonication. The lysate was centrifuged and the clarified supernatant loaded onto a 5ml HisTrap HP nickel affinity column (GE Healthcare). dCRY protein was eluted with 100 mM imidazole, diluted with low salt buffer (50 mM Tris pH 8.0, 5% glycerol, 1mM DTT) and loaded onto a 5 ml DEAE sepharose anion exchange column (GE Healthcare). After gradient elution (0 to 500 mM NaCl), dCRY containing fractions were concentrated and loaded onto a HiLoad S200 16/60 size exclusion chromatography (SEC) column (buffer 25 mM Tris pH 8.0, 150 mM NaCl, 5% glycerol, 1 mM TCEP). SEC fractions containing pure dCRY protein were pooled, concentrated and stored at -80°C until further use. All purification steps were carried out in dark- or dim red light conditions.

UV/VIS spectroscopy of L-Cry and dCRY. UV/VIS absorption spectra of purified L-Cry and dCRY proteins were recorded on a Tecan Spark 20M plate reader. An intensity calibrated naturalistic moonlight source (Fig. S2B) was used for moonlight UV/VIS spectroscopy on L-Cry and dCRY. Naturalistic full moon (FM) intensity was set to 9.67 x 10^{10} photons $cm^{-2}s^{-1}$. To analyze moonlight dose-dependent FAD photoreduction of L-Cry, dark-adapted L-Cry was illuminated with different moonlight intensities (1/3 FM, 1/2 FM, FM and 2 FM intensity) continuously for 4 h on ice and UV-VIS spectra (300 – 700 nm) were collected after 4 h. To analyze sunlight- and moonlight dependent FAD photoreduction of dCRY, dark-adapted dCRY (kept on ice) was continuously illuminated with naturalistic sunlight (1.55 x 10^{15} photons cm^{-2} s^{-1} at the sample) or naturalistic moonlight (9.67 x 10^{10} photons cm^{-2} s^{-1} at the sample) and UV-VIS spectra (300 – 700 nm) were collected at different time points.

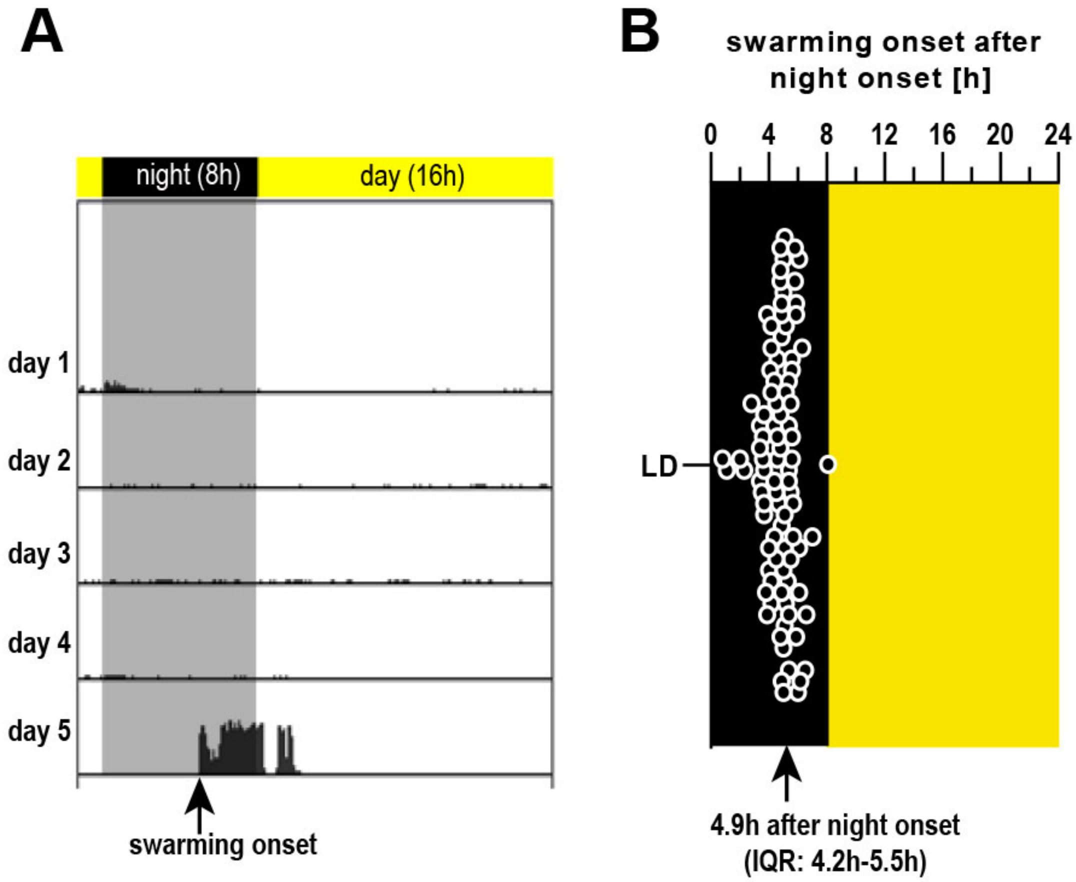

Fig. S1. Determination of the timing of swarming onset by tracking locomotor activity.

(A) Exemplary actogram showing locomotor activity of a sexually maturing worm during the days prior to swarming and in the night of swarming. Swarming onset is correlated with a striking increase in locomotor activity. See **Video S1**. **(B)** Coordinated swarming onset of separated worms that were kept under a 16h:8h LD cycle for at least 9 days prior to swarming (n=92). Median swarming onset was 4.9h after night onset (IQR: 4.2h-5.5h)

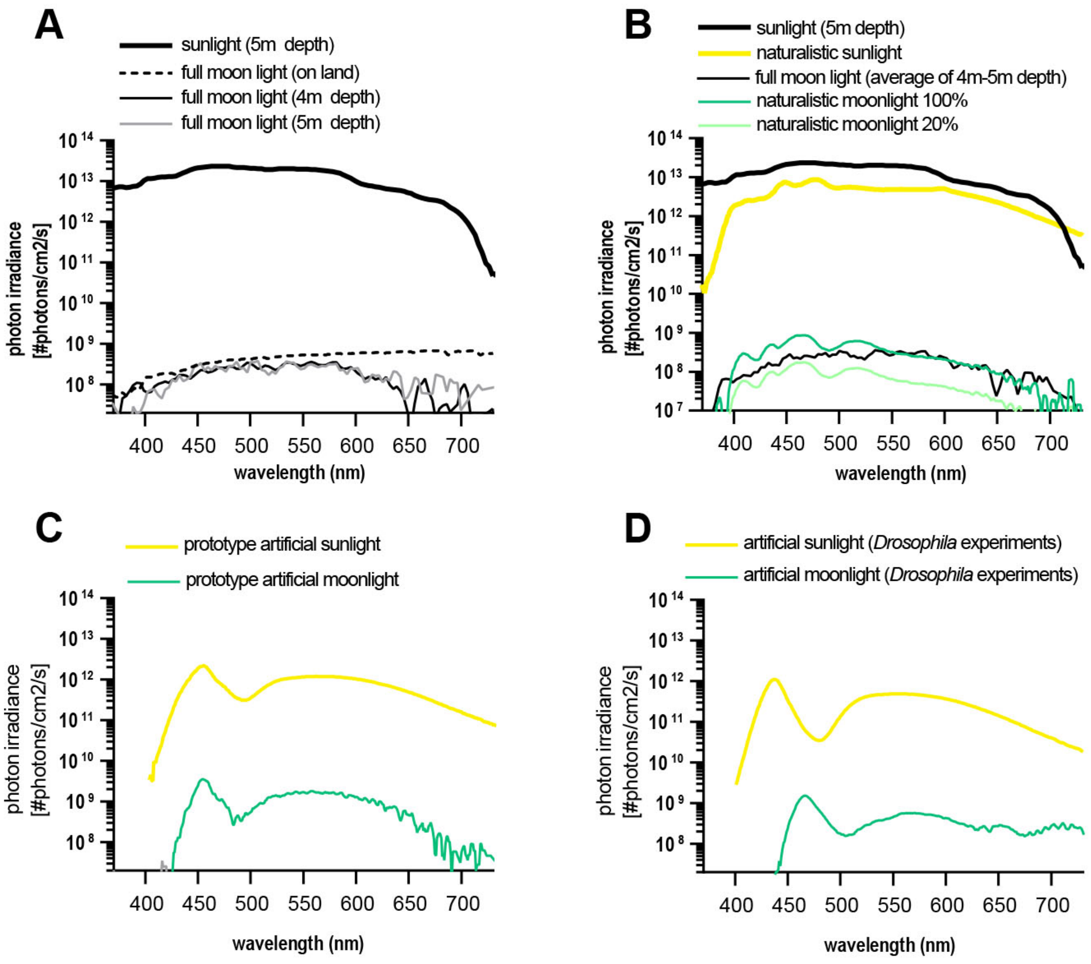

E

light source	photons/cm²/s [380nm-750nm]
sunlight (5m depth)	$4.4x10^{15}$
naturalistic sunlight	$1.3x10^{15}$
prototype artificial sunlight	$2.3x10^{14}$
artificial sunlight (*Drosophila* exp)	$3.5x10^{13}$
full moon light under water (average 4m and 5m depth)	$5.9x10^{10}$
naturalistic moonlight 100%	$9.7x10^{10}$
naturalistic moonlight 20%	$1.9x10^{10}$
prototype artificial moonlight	$6.2x10^{10}$
full moon light on land	$1.8x10^{11}$
artificial moonlight (*Drosophila* exp)	$1.2x10^{11}$

Fig. S2. Sun- and moonlight spectra.

(A) Exemplary natural sunlight and full moon spectra measured under water at the natural *Platynereis* habitat in the coastal waters of Ischia/Italy. Sunlight spectrum was measured at 5m water depth on November 25, 2011 ($9.7x10^{10}$ photons/cm2/s [380nm-750nm], average 10am-4pm), and the two full moon spectra were measured at 4m and 5m water depth on April 17-18, 2012 (average 10:15pm-2am) and November 10-11, 2011 (average 10pm-1am), respectively. To benchmark the underwater moonlight measurements a publicly available full moon light spectrum measured on land is included (http://www.olino.org/blog/us/articles/2015/10/05/spectrum-of-moon-light). **(B)** Spectra of custom-designed naturalistic sun- (yellow) and moonlight (dark and light green) used for all *Platynereis* experiments (except for Fig.1B, 2F,G and **Fig. S1**) compared to natural sun- and moonlight spectra. **(C)** Prototype artificial sun- and moonlight spectra used for experiments shown in Fig. 1B and Fig. 2F,G. **(D)** Artificial sun and moonlight experiments used for Drosophila experiments. **(E)** Total light intensities of the spectra shown in (A-D). All spectra reflect light intensities at the distance relevant for experiments.

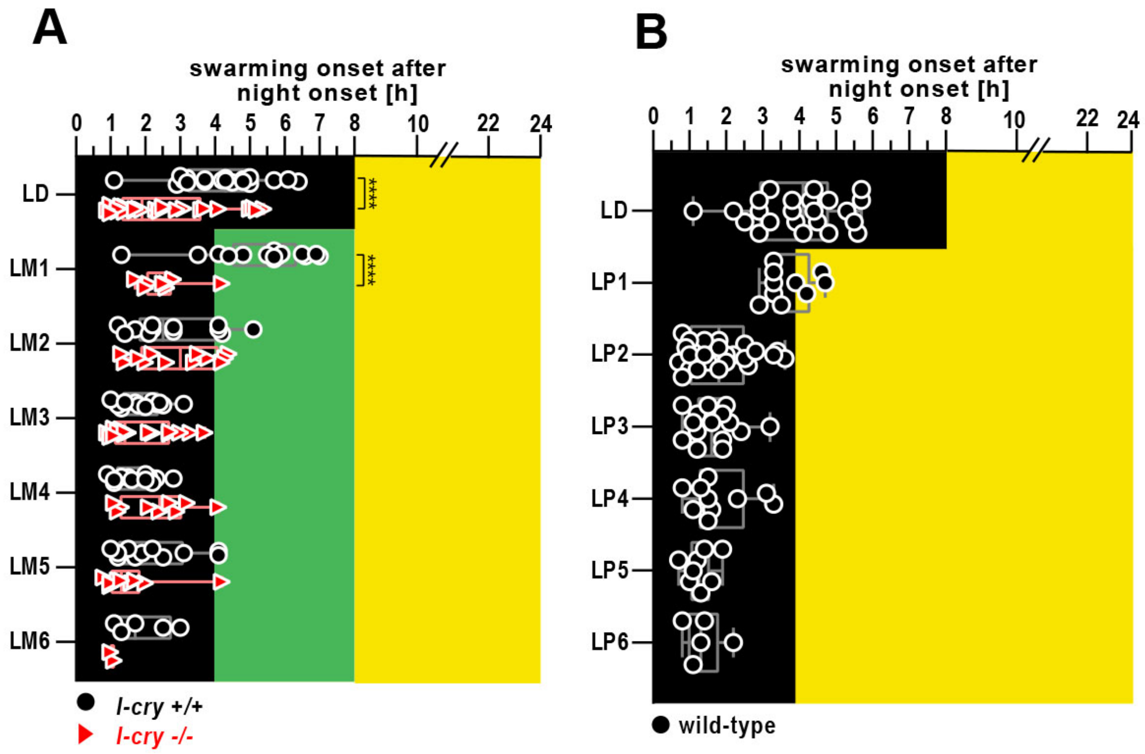

Fig. S3 Comparison of photoperiodic and constant waning moonlight effects on the adjustment of swarming hour.

(A) Swarming onset of wild-types (black circles) and *l-cry*$^{-/-}$ mutants (red triangles) entrained to 16:8h LD cycles and subsequently subjected to 4h of moonlight during the second half of the night **(B)** Swarming onset of wild-types entrained as in (A), but subsequently exposed to additional 4h of sunlight during the second half of the night, mimicking a longer photoperiod (LP).

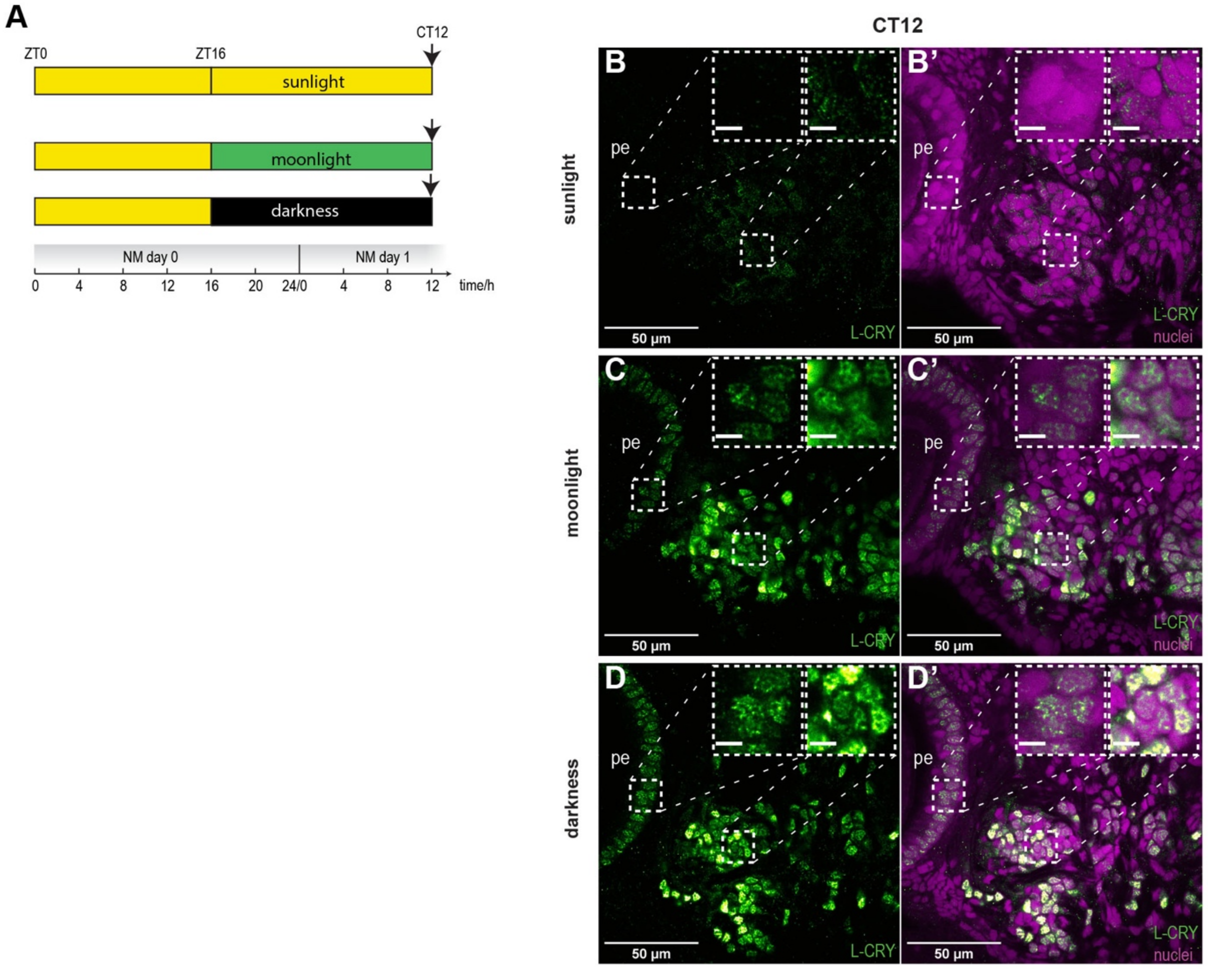

Fig. S4. *Pdu*-L-Cry localization after prolonged darkness, naturalistic sun- or moonlight at CT12. (A) Temporal sampling scheme of *Platynereis* heads. **(B-D´)** Wild-type worm heads sampled under indicated conditions, stained with anti- *Pdu*-L-Cry (green) and the nuclear stain HOECHST (violet). Scale bar in inserts: 5µm. pe=posterior eye.

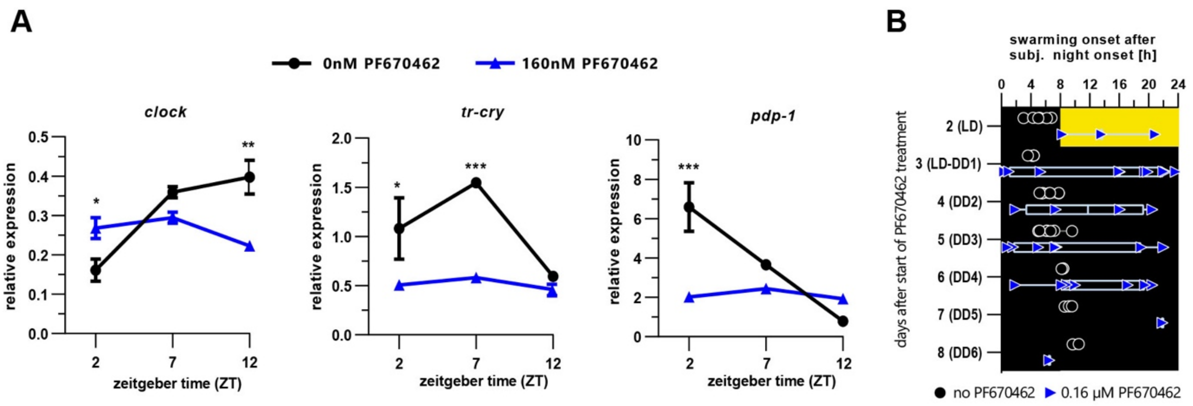

Fig. S5. Treatment with a casein kinase 1δ/ε inhibitor disrupts circadian clock oscillations and desynchronizes swarming onset.

(A) Treatment of 160nM of casein kinase 1δ/ε inhibitor PF670462 results in severely disrupted circadian clock gene transcriptional oscillations in head extracts of premature worms. Expression levels are normalized to *cdc5* transcript levels. **(B)** Swarming onset of worms after at least 9 days after last FM stimulus under LD followed by DD conditions treated with the casein kinase 1δ/ε inhibitor PF670462 (blue triangles); untreated references (black dots) include individuals also shown in Fig. 1C. Values are means ± SEM; n = 3BRs with 4-5 heads/BR. * : p<0.05; ** : p<0.001; *** : p<0.0001 2-way ANOVA followed by Sidak´s multiple comparison test.

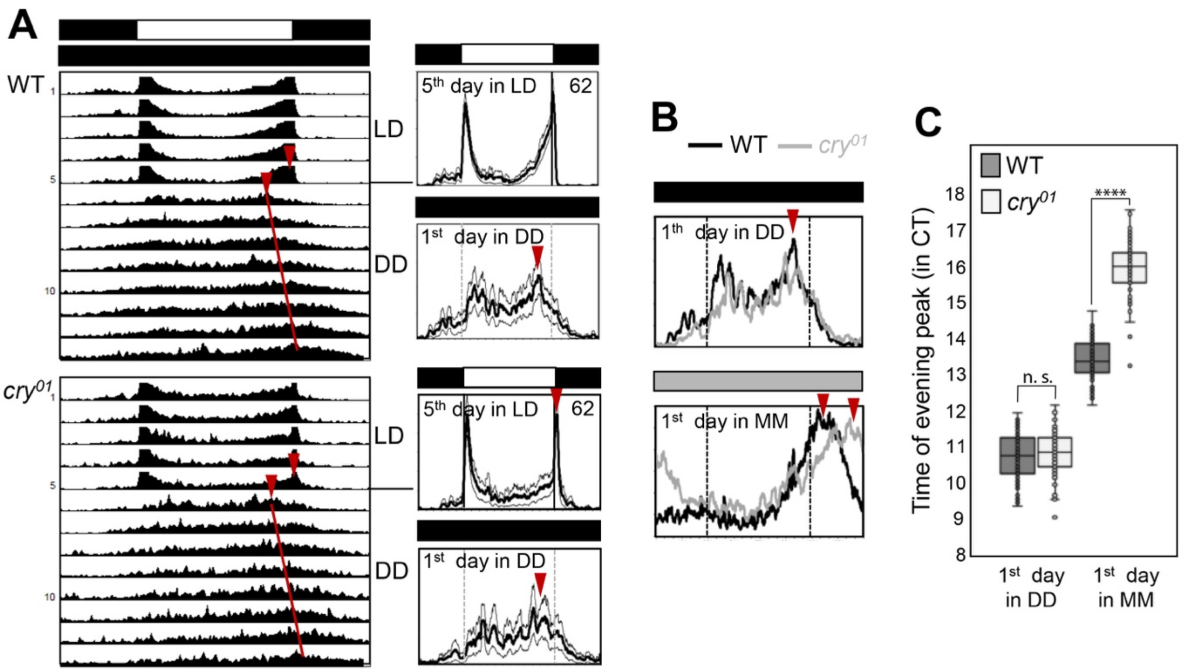

Fig. S6. The delayed evening activity of cry^{01} mutants depends on moonlight.

(A) Actograms and daily profiles depicting average activity of about 60 wild-type (WT) and cry^{01} flies, respectively, under 12:12 light:dark (LD) cycles followed by constant darkness (DD). Timing of evening activity in WT and cry^{01} flies is similar under LD and DD (acrophases of the E-peaks are highlighted by red arrowheads and red lines in the actograms). The average activity profiles shown to the right of the actograms are calculated from the last (5th) day in LD, and the first day in DD, respectively. Thin lines above and below the average lines represent the standard error of the mean and numbers in the diagrams indicate the number of tested flies. **(B)** Comparison of WT and cry^{01} average activity profiles on the first day in DD and MM, respectively. **(C)** Boxplots showing the E-peak acrophases of WT and cry^{01} flies on the first day in DD and MM, respectively. ****$p<0.0001$ for unpaired t-test with Bonferroni correction

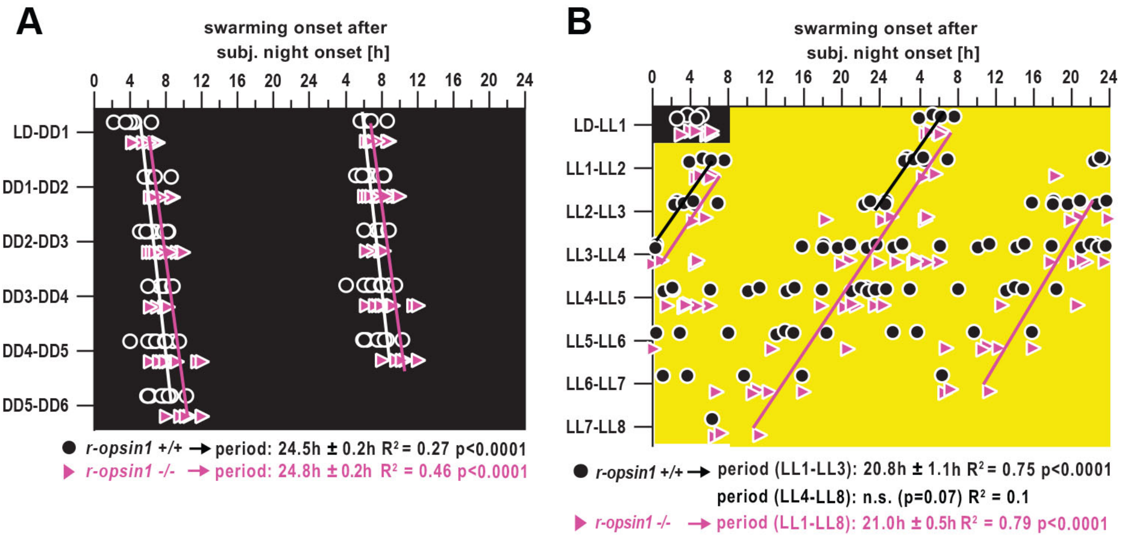

Fig. S7: Time of swarming onset of *r-opsin1*$^{-/-}$ and wt worms under constant conditions.

Recorded swarming time for worms entrained to 16:8h LD cycles and subsequently released into **(A)** constant darkness or **(B)** constant naturalistic sunlight. Black and violet lines indicate linear regression lines of *r-opsin1*$^{+/+}$ and *r-opsin1*$^{-/-}$ worms, respectively. The period length was calculated based on the slope of the regression lines ± the 95% CI of the slope. For discussion on the maintained synchronized swarming and PCC clock advancement in the *r-opsin1* mutants in (B) see **Supplementary Information Text** above.

Movie S1 (separate file). Exemplary video showing mature swarming worms, as well as worms just before swarming

SI References

1. J. Zantke, *et al.*, Circadian and circalunar clock interactions in a marine annelid. *Cell Rep.* **5**, 99–113 (2013).
2. E. Arboleda, *et al.*, Differential impacts of the head on *Platynereis dumerilii* peripheral circadian rhythms. *Front. Physiol.* **10**, 1–14 (2019).
3. H. Ohta, S. Yamazaki, D. G. McMahon, Constant light desynchronizes mammalian clock neurons. *Nat. Neurosci.* **8**, 267–269 (2005).
4. C. P. Coomans, A. Ramkisoensing, J. H. Meijer, The suprachiasmatic nuclei as a seasonal clock. *Front. Neuroendocrinol.* **37**, 29–42 (2015).
5. S. An, *et al.*, A neuropeptide speeds circadian entrainment by reducing intercellular synchrony. *Proc. Natl. Acad. Sci. U. S. A.* **110**, 4355–4361 (2013).
6. J. R. Jones, T. Simon, L. Lones, E. D. Herzog, SCN VIP neurons are essential for normal light-mediated resetting of the circadian system. *J. Neurosci.* **38**, 7986–7995 (2018).
7. R. Revilla-i-Domingo, *et al.*, Characterization of cephalic and non-cephalic sensory cell types provides insight into joint photo-and mechanoreceptor evolution. *Elife* **10**, 1–31 (2021).
8. D. Arendt, K. Tessmar, M.-I. M. de Campos-Baptista, A. Dorresteijn, J. Wittbrodt, Development of pigment-cup eyes in the polychaete *Platynereis dumerilii* and evolutionary conservation of larval eyes in Bilateria. *Development* **129**, 1143–54 (2002).
9. D. Arendt, K. Tessmar-Raible, H. Snyman, A. W. Dorresteijn, J. Wittbrodt, Ciliary photoreceptors with a vertebrate-type opsin in an invertebrate brain. *Science* **306**, 869–871 (2004).
10. J. C. R. Cardoso, M. G. Garcia, D. M. Power, Tracing the origins of the pituitary adenylate-cyclase activating polypeptide (PACAP). *Front. Neurosci.* **14**, 1–19 (2020).
11. P. Bauknecht, G. Jékely, Large-scale combinatorial deorphanization of *Platynereis* neuropeptide GPCRs. *Cell Rep.* **12**, 684–693 (2015).
12. K. Tessmar-Raible, *et al.*, Conserved sensory-neurosecretory cell types in annelid and fish forebrain: insights into hypothalamus evolution. *Cell* **129**, 1389–400 (2007).
13. V. B. Veedin Rajan, *et al.*, Seasonal variation in UVA light drives hormonal and behavioural changes in a marine annelid via a ciliary opsin. *Nat. Ecol. Evol.* **5**, 204–218 (2021).
14. B. Poehn, *et al.*, A Cryptochrome adopts distinct moon- and sunlight states and functions as moonlight interpreter in monthly oscillator entrainment. *bioRxiv* (2021) https:/doi.org/10.1101/2021.04.16.439809.
15. K. Tessmar-Raible, P. R. H. Steinmetz, H. Snyman, M. Hassel, D. Arendt, Fluorescent two-color whole mount in situ hybridization in *Platynereis dumerilii* (Polychaeta, Annelida), an emerging marine molecular model for evolution and development. *Biotechniques* **39**, 460–462 (2005).
16. T. Yoshii, S. Vanin, R. Costa, C. Helfrich-Förster, Synergic entrainment of *Drosophila's* circadian clock by light and temperature. **24**, 452–464 (2009).
17. H. J. Bailes, R. J. Lucas, Human melanopsin forms a pigment maximally sensitive to blue light ($\lambda max \approx 479$ nm) supporting activation of Gq/11 and Gi/o signalling cascades. *Proc. R. Soc. B Biol. Sci.* **280** (2013).
18. A. Berndt, *et al.*, A novel photoreaction mechanism for the circadian blue light photoreceptor *Drosophila* Cryptochrome. *J. Biol. Chem.* **282**, 13011–13021 (2007).
